# Single nuclei RNA sequencing investigation of the Purkinje cell and glial changes in the cerebellum of transgenic Spinocerebellar ataxia type 1 mice

**DOI:** 10.3389/fncel.2022.998408

**Published:** 2022-11-15

**Authors:** Ella Borgenheimer, Katherine Hamel, Carrie Sheeler, Francisco Labrada Moncada, Kaelin Sbrocco, Ying Zhang, Marija Cvetanovic

**Affiliations:** ^1^Department of Neuroscience, University of Minnesota, Minneapolis, MN, United States; ^2^Minnesota Supercomputing Institute, University of Minnesota, Minneapolis, MN, United States; ^3^Institute for Translational Neuroscience, University of Minnesota, Minneapolis, MN, United States

**Keywords:** cerebellum, SCA1, Purkinje cells, Bergmann glia, oligodendrocytes, velate astrocytes

## Abstract

Glial cells constitute half the population of the human brain and are essential for normal brain function. Most, if not all, brain diseases are characterized by reactive gliosis, a process by which glial cells respond and contribute to neuronal pathology. Spinocerebellar ataxia type 1 (SCA1) is a progressive neurodegenerative disease characterized by a severe degeneration of cerebellar Purkinje cells (PCs) and cerebellar gliosis. SCA1 is caused by an abnormal expansion of CAG repeats in the gene *Ataxin1* (*ATXN1*). While several studies reported the effects of mutant ATXN1 in Purkinje cells, it remains unclear how cerebellar glia respond to dysfunctional Purkinje cells in SCA1. To address this question, we performed single nuclei RNA sequencing (snRNA seq) on cerebella of early stage *Pcp2-ATXN1[82Q]* mice, a transgenic SCA1 mouse model expressing mutant ATXN1 only in Purkinje cells. We found no changes in neuronal and glial proportions in the SCA1 cerebellum at this early disease stage compared to wild-type controls. Importantly, we observed profound non-cell autonomous and potentially neuroprotective reactive gene and pathway alterations in Bergmann glia, velate astrocytes, and oligodendrocytes in response to Purkinje cell dysfunction.

## Introduction

Glial cells play key roles required for normal brain function (Barres, 2008). These roles include maintaining homeostasis of ions, neurotransmitters, and water, providing neurotrophic and energy support, removal of unused synapses, and fast propagation of neuronal potentials (Kofuji and Newman, 2004; Parkhurst et al., 2013; Perea et al., 2014; Verkhratsky and Nedergaard, 2018). In most neurodegenerative diseases, glial cells undergo reactive gliosis: a process that includes gene expression, morphological, and functional changes (Burda and Sofroniew, 2014; Escartin et al., 2021). These glial changes have shown an active role in the pathogenesis of neurodegenerative diseases (Lobsiger and Cleveland, 2007; Sheeler et al., 2020). In some situations, reactive gliosis was beneficial by delaying and ameliorating pathogenesis, but reactive glia may also be harmful and can exacerbate neuronal dysfunction and disease pathogenesis (Heneka et al., 2014; Pekny et al., 2014; Kim et al., 2018). The neuroprotective or harmful nature of reactive glia likely depends on many factors including the stage of disease progression, the signaling that triggers gliosis, and glial gene expression changes.

Glia can become reactive in response to external and/or internal signaling. For instance, in all diseases, glia respond to neuronal dysfunction via external/non-cell autonomous signaling. Additionally, in some neurodegenerative diseases, mutant proteins are expressed within glial cells and can contribute to reactive changes in internal/cell-autonomous manner. Investigating internal (cell–autonomous) and external (non-cell-autonomous) reactive glial gene expression changes and their effect on neurons will lead to a better understanding of the pathogenesis of neurodegenerative diseases.

Spinocerebellar ataxia type-1 (SCA1) is a dominantly inherited neurodegenerative disorder caused by the abnormal expansion of CAG repeats in the *Ataxin-1* (*ATXN1)* gene (Orr et al., 1993). Expansion of 39 or more CAG repeats drives severe pathology of cerebellar Purkinje cells and cerebellar gliosis. SCA1 symptoms include loss of balance and coordination, swallowing and speech difficulties, impairments in cognition and mood, and premature death (Orr and Zoghbi, 2007; Jacobi et al., 2013; Rüb et al., 2013; Moriarty et al., 2016; Diallo et al., 2017, 2019). While *ATXN1*-targeting antisense oligonucleotides (ASOs) show promise in pre-clinical trials (Friedrich et al., 2018), there are currently no disease-modifying treatments available for SCA1. The lack of available treatment options highlights the need for increased understanding of SCA1 pathogenesis. This is particularly relevant when considering early disease stages where the development of effective therapies may lead to the delayed onset, reversal, or slowing of disease phenotypes.

Previous studies used mouse models of SCA1 to demonstrate that mutant ATXN1 causes gene expression changes in Purkinje cells. In addition, we have previously shown that glia undergo reactive gliosis in SCA1 mice and contribute to disease pathogenesis in a stage-of-disease dependent manner. Specifically, we found that glia are neuroprotective early and harmful late in SCA1 disease progression, thus suggesting that glial function could be a key therapeutic target for SCA1 (Kim et al., 2018). Our aim in this work was to investigate the underlying changes occurring in glia across the SCA1 cerebellum and, in doing so, to highlight key pathways that may be important for glial responses to PC dysfunction. To accomplish this, we used the transgenic SCA1 mouse model, *Pcp2- ATXN1[82Q]* line which expresses mutant ATXN1 only the cerebellar Purkinje cells (Burright et al., 1995). This allows us to specifically identify the non-cell autonomous pathways activated in glia by dysfunctional Purkinje cells without the confounding factor of gene expression changes driven by mutant ATXN1 expression within glial cells.”

To investigate gene expression changes at the cell-type level we used single-nuclei RNA sequencing. Bulk RNA sequencing has been useful for determining cerebellum-wide changes in the gene expression in SCA1 mouse models (Serra et al., 2004; Ingram et al., 2016a; Driessen et al., 2018). However, bulk RNA sequencing precludes the detection of transcriptional changes at the single-cell level and is also confounded by the possible change in the proportion of cell types (Klein et al., 2015; Lacar et al., 2016; Habib et al., 2017). Previous studies have shown advantages of single nuclei over single cell RNAsequencing. These include preservation of a larger number of cells across multiple subtypes, while minimizing the effects of cell dissociation on gene expression, and enriching for transcripts that are being actively transcribed *in vivo* (Lacar et al., 2016; Habib et al., 2017). Thus, we isolated nuclei from cerebella of SCA1 mice and wild-type controls, and used rigorous analysis to investigate gene expression changes in cerebellar Purkinje cells, and three types of cerebellar glia: Bergmann glia, velate astrocytes, and oligodendrocytes.

## Materials and methods

### Mice

The creation of the *Pcp2-ATXN1[82Q]* mice was previously described (Burright et al., 1995). We have backcrossed these mice onto the C57BL/6 background for 10 generations. As CAG repeats are unstable and tend to shrink in mice, we periodically sequence the CAG region to evaluate number of repeats (Gatchel and Zoghbi, 2005). At the time of experiments the average number of CAG repeats in our colony was 71. We used 12 week old *Pcp2-ATXN1[82Q]* mice and their littermate wild-type control mice. Animal experimentation was approved by the Institutional Animal Care and Use Committee (IACUC) of University of Minnesota and was conducted in accordance with the National Institutes of Health’s (NIH) Principles of Laboratory Animal Care (86–23, revised 1985), and the American Physiological Society’s Guiding Principles in the Use of Animals (National Research Council (Us) Committee for the Update of the Guide for the Care and Use of Laboratory Animals, 2011).

### Nuclei isolation

Nuclei for RNA sequencing were isolated using detergent mechanical lysis protocol as previously described (Matson et al., 2018). Briefly, frozen or fresh whole cerebellum was placed into 1.5mL tube with 500uL pre-chilled detergent lysis buffer [low sucrose buffer (LSB) (sucrose 0.32M, 10 mM HEPES, 5mM CaCl2, 3mM MgAc, 0.1mM EDTA, 1mM DTT) with 0.1% Triton-X] and the tissue was homogenized using a mechanical homogenizer. A 40um strainer was placed over a pre-chilled 50mL tube and pre-wetted with 1ml LSB. 1mL of LSB was added to the tube containing the crude nuclei in the lysis buffer and mixed gently by pipetting (2-3 times). Crude nuclei prep was next passed over the 40uM strainer into the pre-chilled 50mL tube, washed with 1 ml LSB, and centrifuged at 3,200 g for 10 min at 4C.

The pellet was resuspended in 3mL of LSB while gently swirling to remove the pellet from the wall, facilitating resuspension, and left on ice for 2 min. The suspension was transferred to an Oak Ridge tube and nuclei were homogenized in LSB for 15-30 s, all while keeping the sample on ice. Using a serological pipette, 12.5mL of density sucrose buffer (sucrose 1M, 10 mM HEPES, 3mM MgAc, 1mM DTT) was layered underneath the LSB homogenate, taking care not to create a bubble which would disrupt the density layer. Samples were then centrifuged at 3,200 g for 20 min at 4C and pelleted nuclei were resuspended in the resuspension solution (PBS, 0.4 mg/ml BSA, 0.2 U/μl RNAse inhibitor). Nuclei were filtered through a 40um pore-size strainer followed by a 30um and 20um pore-size strainers. A small sample of nuclei was pelleted and added to a slide with VectaShield with DAPI to verify single nuclei isolation under fluorescent microscope (Supplementary Figure 1). The nuclear suspensions were processed by the Genomic Core at the University of Minnesota using 10X Chromium 3′ GEX Capture to Library Preparation (Chromium Next GEM Single Cell 3′ Reagent Kits v3.1 with Single Cell 3′ v3.1 Gel Beads and Chromium Next GEM Chip G).

### Sequencing and analysis

Library quality control was performed using the MiSeq system to estimate average numbers of nuclei per donor mouse. Then all nuclei from 6 donors were multiplexed and sequenced on two independent runs on the Illumina NovaSeq platform, using 1 full lane of S4 chip each time. Sequencing depth ranges from ∼41,000 to ∼120,000 reads per nuclei. Raw, demultiplexed fastq files were analyzed by CellRanger (v5.0.1) using reference genome mm10 (refdata-gex-mm10-2020-A, with the addition of human ATXN1 gene locus), with the option that allows alignment to un-spliced pre-mRNAs (–include-introns). The raw UMI count matrix was cleaned via DIEM algorithm (Alvarez et al., 2020). In summary, DIEM models cell debris using cells with less than 1,000 UMI detected (min_counts = 1,000). In case of overfitting, we increased the threshold to 3,000 (only for the two samples sequenced in the first NovaSeq run). Then the debris scores were manually inspected to exclude true debris. The cleaned gene count table per donor was analyzed with R (v4.1.0) package Seurat (v 4.0.4) for cell type clustering and visualization. Further cell type identification was accomplished with SingleR (v 1.6.1) using DropViz (Saunders et al., 2018). Cerebellum MetaCells reference. We assigned cell types by requiring the same Seurat cluster and the same SingleR annotation.

For identified cell types of Bergmann Cells, Velate Astrocytes, Granule Neurons, Purkinje Neurons, and Oligodendrocytes, scran (v 1.20.1) was used to normalize the single cell expression matrix, followed by limma-trend (v 3.48.3) to test for differential gene expression. Sample batches were considered as an independent factor in the design matrix of the statistical test (design = ∼ genotype + batch). P values were adjusted using Benjamini-Hockberg method. Differential gene expression was determined by an adjusted p-values of 0.05. The lists of differentially expressed genes from different cell types were further analyzed for GO term enrichment using ClusterProfiler (v 4.0.5). Detailed information on the code we used can be found at https://github.com/yingzhang121/snSeq_SCA1.

Visualization of the results was generated using various R packages, including Seurat, ClusterProfiler, enrichplot (v 1.12.2), and ggplot2 (v 3.3.5).

Data is deposited at https://www.ncbi.nlm.nih.gov/geo/query/acc.cgi?acc=GSE215336. To review GEO accession GSE215336 before it becomes public (when results are accepted for publication) please enter token ibqzeqsivrodhkr into the box.

### Immunofluorescent staining

IF was performed on a minimum of six different floating 45-μm-thick brain slices from each mouse (six technical replicates per mouse per region or antibody of interest) as we have previously described (Sheeler et al., 2021). We used primary antibody against DNER (Invitrogen, PA5-99872). Confocal images were acquired using a confocal microscope (Olympus FV1000, Leica Stellaris 8) using a 20X oil objective. Z-stacks consisting of twenty non-overlapping 1-μm-thick slices were taken of each stained brain slice per brain region (i.e., six z-stacks per mouse, each taken from a different brain slice). The laser power and detector gain were standardized and fixed between mice, and all images for mice within a cohort were acquired in a single imaging session to allow for quantitative comparison. To quantify relative intensity of staining we measured the average signal intensity in the region of interest that included PCs soma and dendrites.

### Reverse transcription and quantitative polymerase chain reaction (RTqPCR)

Total RNA was extracted from dissected mouse cerebella, medulla, and hippocampus using TRIzol (Life Technologies), and RT-qPCR was performed as described previously (Lobsiger and Cleveland, 2007). We used IDT Primetime primers for the following genes: *Ptch2*, *Dner* and *Gli*. Relative mRNA levels were calculated using 18S RNA as a control and wild-type mice as a reference using 2^–ΔΔ^*^Ct^* as previously described (Lobsiger and Cleveland, 2007).

### Statistics

**FIGURE 1:**
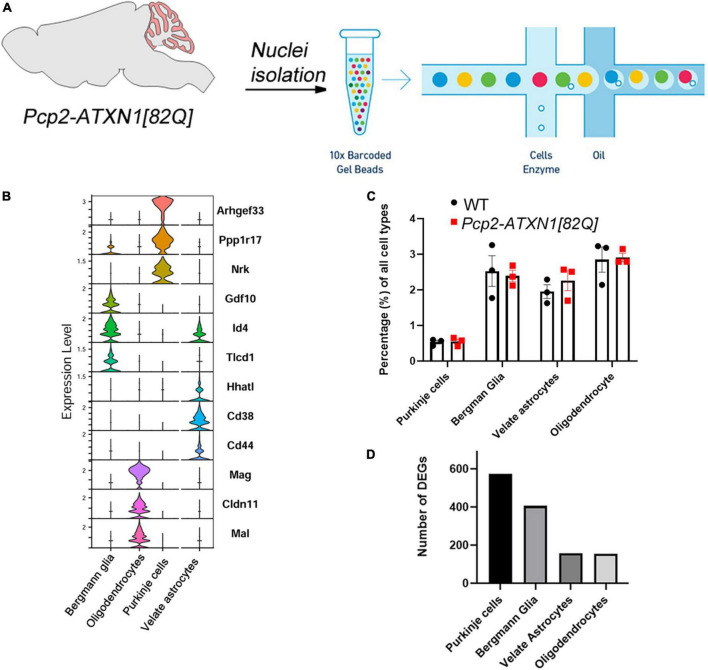
Experimental schematics, cell composition and DEGs per cell type in *Pcp2*-*ATXN1[82Q]* mice. **(A)** Schematic pipeline of SCA1 snRNA-seq and analysis of mouse (*N* = 6 mice) cerebellum (WT *N* = 3, *Pcp2- ATXN1[82Q]* SCA1 *N* = 3). Individual nuclei were isolated from the cerebella of 12 weeks old *Pcp2- ATXN1[82Q]* mice and wild-type littermate controls (*N* = 3 of each). After passing library QC nuclei were sequenced on Illumina NovaSeq platform. **(B)** Normalized violin plots showing expression of cell type-specific marker genes for each mouse cluster to evaluate cell type annotation. 47,894 high-quality, snRNAseq profiles were imported into Seurat for clustering analysis and nuclei were classified into the cell types with SingleR (v 1.6.1) using DropViz Cerebellum MetaCells reference. **(C)** Relative proportions of cell types in WT and SCA1 mice. Average percentages of Purkinje cells (PC), Bergman Glia (BG), velate astrocytes (VA), and oligodendrocytes in *Pcp2- ATXN1[82Q]* mice and wild-type littermate controls (*N* = 3 of each) were calculated as percentage of total cells. Data is presented as mean ± SEM with average values for each mouse represented by a dot. Two-way ANOVA with Sidak’s multiple comparison did not detect any significant difference between *Pcp2- ATXN1[82Q]* mice and wild-type littermate controls (Supplementary Table 2). **(D)** Total number of differentially expressed genes (DEG) in each cell type. *P* values were adjusted using Benjamini-Hockberg method. Significant differential gene expression was determined by an adjusted *p*-values ≤ 0.05.

Wherever possible, sample sizes were calculated using power analyses based on the standard deviations from our previous studies, significance level of 5%, and power of 90%. For RNA sequencing data, limma-trend (v 3.48.3) was used to test for differential gene expression. Sample batches were considered as an independent factor in the design matrix of the statistical test (design = ∼ genotype + batch). P values were adjusted using Benjamini-Hockberg method. Differential gene expression was determined by an adjusted p-values of 0.05. For all other results, statistical tests were performed with GraphPad Prism 7.0 (GraphPad Software Inc.). Data was analyzed using unpaired Student’s t-test with Welch’s correction (RTqPCR of cerebellar mRNA from wild-type and *Pcp2-ATXN1[82Q]* mice in Supplementary Figures 2, 4A, 6B, and intensity of DNER staining in Supplementary Figure 4D), and two-way ANOVA (cell type and genotype) followed by the Sidak’s multiple comparison test (relative abundance of each cell type in wild-type and *Pcp2-ATXN1[82Q]* mice, Figure 1C) or Tukey’s multiple comparison test (percentage of cerebellar cell types expressing endogenous mouse *Atxn1[2Q]* or transgenic human *ATXN1[82Q]* in wild-type and *Pcp2-ATXN1[82Q]* mice, Supplementary Figure 3). Outliers were determined using GraphPad PRISM’s Robust regression and Outlier removal (ROUT) with a Q = 1% for non-biased selection. Raw data and exact p values are provided in Supplementary Tables 1–4.

## Results

### Mutant ATXN1 expression in Purkinje cells does not alter proportions of cerebellar cells

In this study, we sought to determine the non-cell autonomous reactive gene expression changes in cerebellar glia in response to mutant ATXN1-driven Purkinje cell dysfunction. To ensure that glial cells are affected solely in response to neuronal dysfunction and not because of cell-autonomous expression of expanded ATXN1, we have used a Purkinje cell specific transgenic mouse model of SCA1, *Pcp2-ATXN1[82Q]* mice (Burright et al., 1995). In these mice, mutant *ATXN1* with 82 CAG repeats is selectively expressed under *Purkinje cell protein 2 (Pcp2)* promoter in cerebellar Purkinje cells. We chose to examine glial changes at 12 weeks of age to capture an early stage of SCA1 pathology when disease is still reversible (Zu et al., 2004) and as such therapeutically noteworthy.

Previous bulk RNA sequencing studies showed significant gene expression changes underlying PC dysfunction in the cerebella at 12 weeks, but no detectable PC death (Ingram et al., 2016b; Mellesmoen et al., 2018). We have confirmed that many of these genes, which are considered representative of Purkinje cell pathology in SCA1 by the field, are changed in cerebella of our mice at this time point. These include *Calbindin 1(Calb1)*, *Purkinje cell protein 4* (*Pcp4)*, *Regulator of G protein signaling (Rgs8)*, *inositol 1,4,5-trisphosphate receptor* (*ITPR)*, *inositol polyphosphate-5-phosphatase (Inpp5)* and *GTPase Activating Rap/RanGAP Domain Like 3 (Garnl3)* (Ingram et al., 2016b). All of these genes were significantly downregulated in our 12 week old mice, indicating that molecular aspects of mutant ATXN1 on PCs were easily detectable in bulk tissue (Supplementary Figure 2 and Supplementary Table 1).

To investigate the gene expression changes attributable to specific cell types, we isolated individual nuclei from the cerebella of six 12 week old SCA1 and littermate control mice (Figure 1A and Supplementary Figure 2). Nuclei were captured and barcoded followed by single nuclei RNA sequencing (snRNAseq, Figure 1A; Klein et al., 2015; Zilionis et al., 2017). After removing debris nuclei using semi-supervised machine learning classifier Debris Identification using Expectation Maximization (DIEM) (Alvarez et al., 2020), we identified a total of 47,894 high-quality, snRNAseq profiles. They were imported into Seurat for clustering analysis and visualization. We then classified nuclei into the cell types with SingleR (v 1.6.1) using DropViz Cerebellum MetaCells reference (Saunders et al., 2018; Figure 1B).

First, we wanted to determine whether cellular composition of the cerebellum is altered in SCA1, e.g., whether expression of mutant ATXN1 in Purkinje cells leads to the relative increase or decrease in the numbers of the main cerebellar cell types, including Purkinje cells (PCs), Bergmann glia (BG), velate astrocytes (VA) and oligodendrocytes (OL). We calculated the percentage of each of these cell types by dividing the number of nuclei identified as a specific cell type by the total number of nuclei. Comparing *Pcp2-ATXN1[82Q]* and wild-type mice, we have not found statistically significant difference in the relative percentages of Purkinje cells (0.55 ± 0.068 vs. 0.53 ± 0.052), Bergmann glia (2.39 ± 0.16 vs. 2.52 ± 0.43), velate astrocytes (2.26 ± 0.28 vs. 1.95 ± 0.19), nor oligodendrocytes (2.91 ± 0.12 vs. 2.85 ± 0.35) indicating that at this early disease stage we cannot detect a change in the cerebellar cell type composition in *Pcp2-ATXN1[82Q]* mice (Figure 1C and Supplementary Table 2).

Additionally, we determined which cerebellar cells express wild-type mouse and mutant human *ATXN1.* We found that endogenous wild-type mouse *Atxn1[2Q]* is expressed in PCs, BG, VA, and OL (Supplementary Figure 3A and Supplementary Table 3). Furthermore, mutant human *ATXN1[82Q]* is expressed only in PC cells in *Pcp2- ATXN1[82Q]* mice, confirming the Purkinje cell expression specificity of this line (Supplementary Figure 3B and Supplementary Table 4). Additionally, no mutant human *ATXN1* expression was found in any cell type in wild-type littermate control mice.

### Selective expression of mutant ATXN1 in Purkinje cells causes significant gene expression changes in cerebellar glia

Most gene expression studies to date have focused on understanding how mutant ATXN1 affects Purkinje cells. Here we confirmed that many of the genes considered representative of Purkinje cell-specific pathology are indeed uniquely changed in Purkinje cells. In addition, we investigated gene expression changes in BG, VA and OL in response to PC pathology. Using differential gene expression (DEG) analysis, we identified genes altered in each of these four cell types. We found that each cell type is uniquely affected by ATXN1-driven PC dysfunction and demonstrate different numbers of DEGs (Figure 1D). Remarkably, BG and PCs had a comparable number of DEGs (575 and 406, respectively) at 12 weeks despite the fact that mutant ATXN1 is expressed only in PCs in these mice. These significant non-cell autonomous transcriptional alterations in BG may suggest that BG are highly sensitive to perturbation in PCs. Additionally, VA and OL had a notable number of DEGs (157 and 155, respectively, Figure 1D). These results indicate that BG, VA and OL all respond to mutant ATXN1 induced dysfunction in PCs.

### Single-nuclei analysis confirms previously identified gene expression changes in Purkinje cells and identifies novel and potentially compensatory genes

Previous studies described different sub-populations of PCs based on gene expression profiles and cellular physiology (Zhou et al., 2014; Kozareva et al., 2020). Moreover, several studies have indicated that SCA1 pathology is not uniform across the cerebellum (White et al., 2021). However, most of our understanding of mutant ATXN1 gene expression changes in Purkinje cells is derived from bulk RNA sequencing analysis. This prevents any identification of altered pathology in different sub-types of Purkinje cells which may be more vulnerable or disease resistant (Serra et al., 2004; Ingram et al., 2016b). To address this, we analyzed gene expression changes in PCs and compared the results to previously reported transcriptional alterations in SCA1.

**FIGURE 2:**
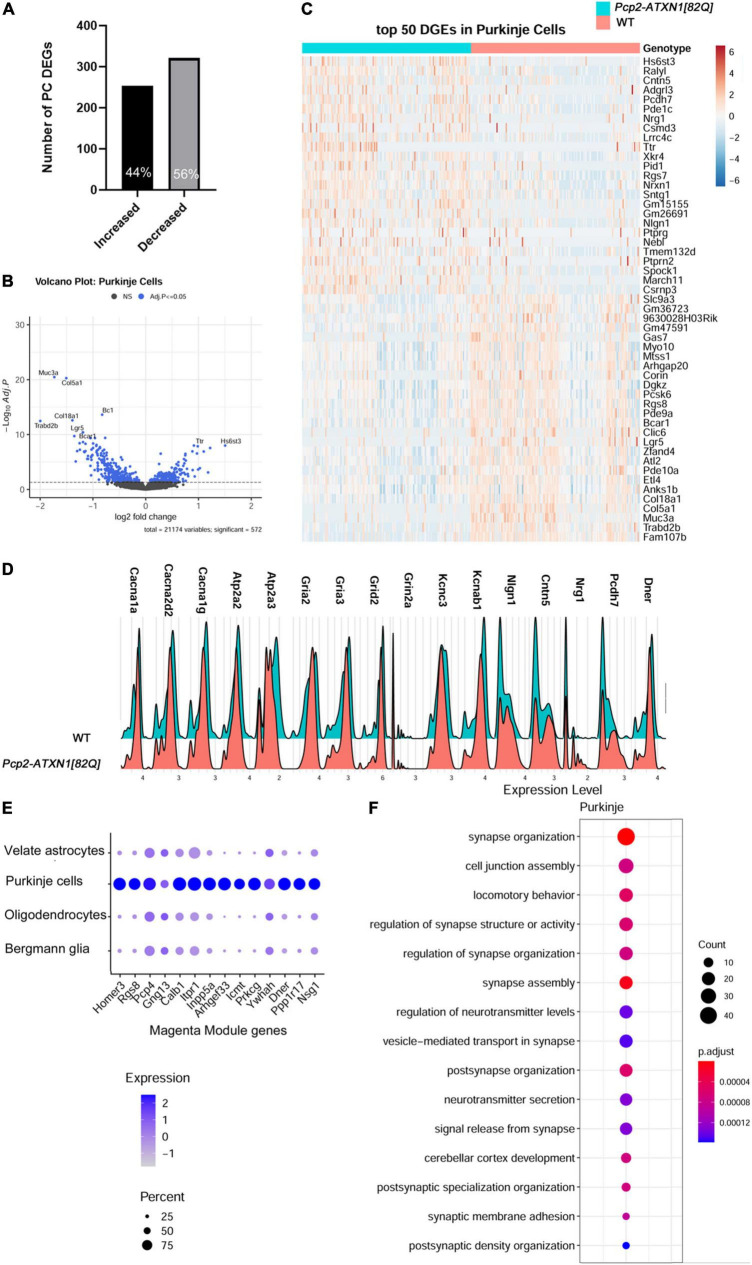
Single nuclei analysis identifies SCA1 disease associated transcriptional changes in Purkinje cells. For identified Purkinje Neurons, limma was used to test differential gene abundance between control and *Pcp2-ATXN1[82Q]* samples. *N* = 3 mice of each genotype. *P* values were adjusted using Benjamini-Hockberg method. Differential gene expression was determined by an adjusted p-values of 0.05. **(A)** Number of upregulated and downregulated differentially expressed genes (DEGs) in PCs. **(B)** Volcano plot showing DEGs of PC cluster in *Pcp2-ATXN1[82Q]* SCA1. **(C)** Heatplot displaying expression profiles of top 25 highest upregulated and downregulated PC DEGs in wild-type and *Pcp2-ATXN1[82Q]* samples determined by logFC values with adjusted *p*-values ≤0.05. *N* = 3 mice of each genotype. **(D)** Ridgeplot showing distribution of expression of selected genes in wild-type and *Pcp2-ATXN1[82Q]* Purkinje cells. **(E)** Dot plot of the differential expression of Magenta genes in Purkinje cells, Bergmann glia, velate astrocytes and oligodendrocytes with color of dot depicting expression and size of the dot depicting percentage of cells. All selected genes had adjusted *p*-value ≤ 0.05. **(F)** Pathway analysis of total DEGs between WT and *Pcp2-ATXN1[82Q]* PCs at 12 weeks of age. The color of dots depicts the adjusted *p* values, radius of the dot depicts gene counts (number of genes in the enriched pathway).

We identified 575 DEGs in Purkinje cells (false discovery rate adjusted p value p.adj/q < 0.05), 56% of which were downregulated and 44% upregulated (Figure 2A). This prevalence of downregulated genes is consistent with previous results and is generally thought to reflect the direct repressive effect of mutant ATXN1 on PC gene transcription (Ingram et al., 2016b).

Among the top ten upregulated genes were genes known for their roles in neurodevelopment including *Heparan Sulfate 6-O-Sulfotransferase 3* (*Hs6st3*), *Adhesion G Protein-Coupled Receptor L3* (*Adgrl3*), *Contactin 5* (*Cntn5*), and *Neuregulin 1* (*Nrg1*) (Mei and Xiong, 2008; Cocchi et al., 2016; El Hajj et al., 2017; Moon and Zhao, 2021). In addition, SCA1 PCs had increased expression of *RALY RNA Binding Protein-like (Ralyl)*,which is known as an Alzheimer’s disease cognitive reserve gene (Zhang et al., 2020) (Figures 2A–C).

We also found increased expression of synaptic vesicle genes encoding for vacuolar V-type ATP-ase subunits (*Atp6v0c*, *Atpgv0a1*, and *Atp6v1h*) that provide energy in the form of ATP to fuel transport of neurotransmitters into synaptic vesicles and *Solute carrier family 32a1 (Slc32a1)*, encoding vesicular GABA transporter VGAT (Vesicular GABA and Glycine Transporter). Increased expression of genes providing energy for GABA transport into vesicles as well as increased expression of vesicular GABA transporter could potentially result in higher GABA content per vesicle and increased GABA release from SCA1 PCs.

Previous work has demonstrated marked changes in PC firing in SCA1 which indicate dysfunctions in signaling and cellular physiology (Bushart et al., 2021). In keeping with this, we observed a proportion of gene expression changes suggestive of PCs reduced ability to respond to stimuli. This is indicated by the downregulated expression of genes encoding extracellular matrix proteins (*Mucin3a* (*Muc3a)*, *Collagen type 5 alpha1 (Col5a1)*, *Collagen Type 8 Alpha 1 (Col18a*) (Tsai et al., 2021), proteins involved in Wnt and GPCR signaling (*leucine rich repeat containing G protein-coupled receptor 5 (Lgr5)* that enhances Wnt signaling and *phosphodiesterase 10 (Pde10)* that plays a role in signal transduction by regulating the intracellular concentration of cyclic nucleotides cAMP and cGMP) (Carmon et al., 2012), signaling scaffolding protein *Breast cancer anti-estrogen resistance protein 1 (Bcar1)* (Furuichi et al., 2011; Maria del Pilar et al., 2017), and small brain specific non-coding RNA BC1 that regulates dendritic translation (Zhong et al., 2009; Robeck et al., 2016). This perturbed communication of SCA1 PCs is further supported by altered expression of genes involved in calcium and glutamatergic signaling. These downregulated genes encode for calcium voltage gated channels Cav2.1 and Cav3.1 (*calcium voltage-gated channel subunit alpha1 A (Cacna1a), Cacna2d2*, and *Cacna1g*), calcium endoplasmic reticulum pumps SERCA1 and SERCA2 (*ATPase Sarcoplasmic/Endoplasmic Reticulum Ca^2 +^ Transporting 2 (Atp2a2)* and *Atp2a3*), ionotropic glutamate receptors (*glutamate ionotropic receptor AMPA type subunit 2 (Gria2), Gria3, glutamate Ionotropic Receptor Delta Type Subunit 2 (Grid2), Grid2 interacting protein(Grid2ip), glutamate ionotropic receptor NMDA type subunit 2A* (*Grin2a)*, and potassium channels (*potassium Voltage-Gated Channel Subfamily C Member 3* (*Kcnc3)*, associated with SCA13, and *potassium voltage-gated channel subfamily A regulatory beta subunit 1* (*Kcnab1)* (Matsuda et al., 2006; Figures 2C–D). These results are consistent with previous studies implicating ion channel, calcium and potassium dysregulation and synaptic dysfunction in SCA1 (Bushart and Shakkottai, 2018). Notably, several of these genes have also been associated with other ataxias including Spinocerebellar ataxia autosomal recessive 18 (*Grid2)* and Episodic Ataxia Type 1 (*Kcnab1).*

SCA1 is a progressive disease worsening with aging. Previous studies used weighted Gene Co-expression Network Analysis (WGCNA) of cerebellar bulk RNA sequencing data from *Pcp2-ATXN1[82Q]* mice to identify a specific module (the Magenta Module) as a gene network significantly correlated with SCA1 disease progression in Purkinje cells (Ingram et al., 2016b). Notably, of the 342 genes identified in the magenta module, the Allen brain atlas suggested that 94 (27%) are PC enriched, 175 genes (51%) are PC exclusive, and 31 genes (9%) belong to multiple cell types (Ingram et al., 2016b). Using single nuclei gene expression analysis, we determined that 103 (30.1%) of the previously identified Magenta Module genes are altered at 12 weeks of age in *Pcp2-ATXN1*(O’Leary et al., 2020) mice. This is consistent with previous bulk RNA sequencing studies that showed that not all of the Magenta Module genes were found to be significantly altered at 12 weeks in *Pcp2-ATXN1*(O’Leary et al., 2020) mice (Ingram et al., 2016b). Of these genes, 74 were exclusively altered in PCs (71.8%), while 29 (28.2%) were also changed in two or more cell types, including BG, VAs and OLs. Most intriguing were 13 Magenta Module genes that were changed in all four cell types (Figure 2E). For instance, expression of Magenta genes *Itpr1, Pcp4 and Gng13* was significantly suppressed in PCs and, to a lesser extent, in BG, VAs and OLs. As these genes, respectively, encode for proteins regulating calcium and GPCR signaling (such as inositol 3 phosphate receptor 1 (*ITPR1*) that regulates calcium release from the ER, Purkinje Cell Protein 4 (*PCP4*) that regulates calcium binding to calmodulin, and G Protein Subunit Gamma 13 (*Gng13*) that regulates G protein coupled receptor signaling), this indicates that calcium and G protein signaling are perturbed not only in PCs, as previously described, but also in BG, VAs, and OLs.

Among the genes altered in PCs, but not changed in BG, VAs, or OLs, was *Delta/Notch like EGF Repeat containing* (*Dner*). *Dner* has been previously found to be an important component of PC-BG communication whereby cell surface expression of DNER in Purkinje cells induces morphological differentiation and functional maturation of Bergmann glia. Moreover, loss of *Dner* impairs mouse motor behavior implicating its importance for cerebellar function (Eiraku et al., 2005; Tohgo et al., 2006). Reduced *Dner e*xpression could be a mechanism by which SCA1 PCs signal changes to BG. Using RT-qPCR, we confirmed decrease in *Dner* mRNA expression in SCA1 cerebella (41% decrease compared to WT controls, Supplementary Figure 4A and Supplementary Table 1). Furthermore, using immunohistochemistry, we found reduced levels of DNER protein in soma and dendrites of Purkinje cells in *Pcp2-ATXN1[82Q]* mice (0.679 ± 0.006 vs. 0.553 ± 0.041, ∼19% decrease compared to WT controls, Supplementary Figures 4B–D and Supplementary Table 1).

Using hierarchical enriched pathways analysis of up and downregulated genes in PCs we identified pathways involved in synapse function, including synapse organization, cell junction assembly, regulation of synapse structure or activity, synapse assembly, and regulation of neurotransmitter levels (Figure 2F). Among top Gene ontology (GO) pathways associated with the *upregulated* genes were synapse assembly (*q* = 3.42 × 10^–4^), synaptic membrane adhesion (*q* = 6.1 × 10^–4^), cell junction assembly (*q* = 8.8 × 10^–4^), synapse organization (*q* = 3.2 × 10^–3^) and cell junction organization (*q* = 8.1 × 10^–3^). Kyoto Encyclopedia of Genes and Genomes (KEGG) identified axon guidance (*q* = 1.4 × 10^–5^) and synaptic vesicle cycle (*q* = 2.9 × 10^–3^) as altered pathways. GO pathway analysis of *downregulated* genes identified calcium binding (*q* = 9.8 × 10^–3^), and dendrite and dendritic tree (*q* values of 1.45 and 1.48 × 10^–5^, respectively), while top KEGG pathways of downregulated genes were long-term depression (*q* = 4.9 × 10^–4^), cholinergic (*q* = 3.2 × 10^–3^), glutamatergic (*q* = 3.3 × 10^–3^), serotoninergic (*q* = 5.2 × 10^–3^) and dopaminergic (*q* = 5.5 × 10^–3^) synapse and retrograde endocananbinoid signaling (*q* = 7.1 × 10^–3^). Alterations in calcium and glutamatergic signaling are consistent with previous results from bulk RNAseq in SCA1 cerebella. Our results build upon those previous studies by confirming that ATXN1 is driving these changes specifically in PCs. Moreover, our results indicate upregulation of pathways associated with synapse formation as potential compensatory pathways for signaling disruptions in SCA1 PCs.

Notably, most of the observed gene expression changes seem to be present only in a portion of PCs (Figures 2C,D). Consistent with previous studies that suggested intracerebellar differences in the expression of *hATXN1* and in PCs pathology in SCA1 mice (Chopra et al., 2020; Kozareva et al., 2020; White et al., 2021), our analysis indicated that only portion of PCs (∼ 25%) express detectable levels of mutant human *ATXN1* mRNA in this line (Supplementary Figure 3B). We named these cells *hATXN1*+ PCs, while the cells in which we did not detect human ATXN1 we named *hATXN1-* PCs. It is possible that our analysis may underestimate the number of *hATXN1*+ PCs by not detecting low human ATXN1 expression. With that caveat in mind, we next analyzed gene expression changes in SCA1 *hATXN1*+ PCs (31 cells) and *hATXN1-* PCs (110 cells) compared to PCs from wild-type mice (132 cells).

In this limited analysis, we could only identify a handful of DEGs (35) between *hATXN1*+ and *hATXN1-* PCs with at most 1.6 fold change. We have also identified 531 DEGs in *hATXN1*+ PCs and 370 DEG in *hATXN1-* PC cells compared to WT. Notably, 76.5% of the DEG in *hATXN1*+ PCs were upregulated, e.g., out of 531 DEGs 406 genes were upregulated (Supplementary Figure 5 and Supplementary Table 5). All of the 125 downregulated *hATXN1*+ PCs genes were also downregulated in *hATXN1-* PCs. However, out of the 406 genes that were upregulated in *hATXN1*+ PCs, only 47 were upregulated in *hATXN1-* PCs. Among shared upregulated and downregulated genes were previously mentioned top DEGs (Figures 2B,C), but the relative fold change in their expression was larger in *hATXN1*+ PCs (Supplementary Figure 5B and Supplementary Table 5). For instance, log2FC for *Hs6st3*, and *Ralyl* were 2.4 and 1.7 in hATXN1+ *PCs*, and 1.2 and 1.08 in *hATXN1-* PCs. Among upregulated genes that were unique to *hATXN1*+ PCs was *Gabrg2* (*gamma-aminobutyric acid type A receptor subunit gamma2*), which encodes for ionotropic GABA receptor A subunit.

In conclusion, we confirmed changes in PC gene expression previously found using bulk RNA sequencing. These included a number of genes underlying pathways associated with changes in ion transport and synapse function in SCA1 Purkinje cells. We also identified novel genes that deepen our understanding of altered signaling responsiveness of SCA1 PCs. Moreover, we identified novel genes that are upregulated in PCs early in disease progression. These genes may provide compensatory roles, including added support to extracellular matrix underlying synapse structures and added support to signaling strength, such as the increased expression of synaptic vesicle gene *Slc32a1* which may restore inhibitory/excitatory balance in the cerebellar network. Intriguingly, we showed that PCs differ in the expression of mutant ATXN1 and that PCs expressing the highest, and therefore most detectable, levels of mutant ATXN1 show a high degree of gene upregulation. This is in stark contrast to the *hATXN1-* PCs from SCA1 cerebella with undetectable levels of mutant *ATXN1* or total PCs in which most DEGs are downregulated.

### Disrupted Bergmann glia-Purkinje cell signaling via sonic hedgehog in Spinocerebellar ataxia type 1

Bergmann glia are a special type of radial astrocytes which are intimately connected with Purkinje cells, both structurally and functionally. BG cell bodies surround the somas of PCs and their radial processes envelop PCs synapses in the molecular layer. This close morphological interaction is mirrored in the functional interdependence of BG and PCs. BG are essential for PCs function and viability through their many roles including maintenance of potassium homeostasis, removal of synaptic glutamate and provision of neurotrophic support (Rothstein et al., 1996; Grosche et al., 1999; Custer et al., 2006; Djukic et al., 2007; Miyazaki et al., 2017). Similarly, PCs signaling is important for the ongoing function of BG. In particular, sonic hedgehog (Shh) signaling from PCs to BG drives the development and maintenance of Bergmann glia character (Eiraku et al., 2005; Farmer et al., 2016).

With such close interactions, BG are perfectly poised to sense and respond to PC dysfunction in SCA1. Indeed, we have previously shown both that BG undergo reactive gliosis in patients with SCA1 and in SCA1 mouse models (Cvetanovic et al., 2015; Rosa et al., 2021) and that they contribute to disease pathogenesis in SCA1 mice. Thus, we next wanted to investigate molecular changes in BG that underlie their response to PC dysfunction early in SCA1 progression.

**FIGURE 3:**
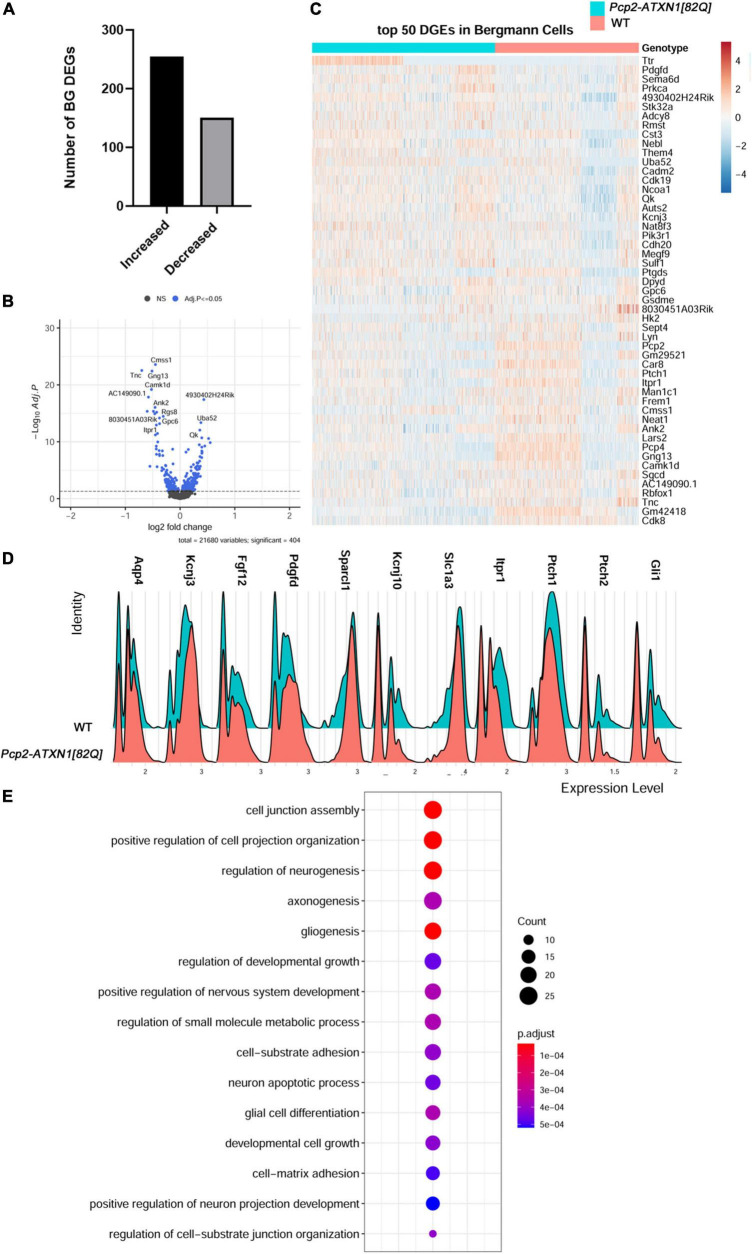
Mutant ATXN1 causes significant non-cell autonomous gene expression perturbations in SCA1 Bergmann glia. **(A)** For identified Bergmann glia, limma was used to test differential gene abundance between control and *Pcp2-ATXN1[82Q]* samples. *N* = 3 mice of each genotype. *P* values were adjusted using Benjamini-Hockberg method. Differential gene expression was determined by an adjusted *p*-values of 0.05. **(A)** Number of upregulated and downregulated differentially expressed genes (DEGs) in BG. **(B)** Volcano plot showing DEGs of BG cluster in *Pcp2-ATXN1[82Q]* SCA1. **(C)** Heatplot displaying expression profiles of top 25 highest upregulated and downregulated DEGs in wild-type and *Pcp2-ATXN1[82Q]* Bergmann glia determined by logFC values with adjusted *p*-values ≤ 0.05. *N* = 3 mice of each genotype. **(D)** Ridgeplot showing distribution of expression of selected genes in wild-type and *Pcp2-ATXN1[82Q]* Bergmann glia. **(E)** Dot plot of the pathway analysis of total DEGs between WT and *Pcp2-ATXN1[82Q]* Bergmann glia at 12 weeks of age. The color of dots depicts the adjusted *p* values, radius of the dot depicts gene counts (number of genes in the enriched pathway).

We identified 406 DEGs (p.adj < = 0.05) in Bergmann glia. Out of these 151 (37.2%) were downregulated and 255 (62.8%) upregulated (Figure 3A).

G*lial fibrillary acidic protein* (*Gfap*) expression was increased, indicating a reactive glial phenotype similar to what we have found in previous work. In addition, we have found increased expression of several genes that have been attributed neuroprotective roles including *secreted protein acidic and rich in cysteine like 1 (Sparcl1), fibroblast growth factor 12 (Fgf12)*, *platelet derived growth factor D (Pdgfd)*, and *Cystatin 3* (*Cst3)* (Figures 3B,C). SPARCL1 promotes excitatory synapse formation. One of the early signs of SCA1 is loss of excitatory VGLUT2+ synapses on PCs (Kim et al., 2018). Increased *Sparcl1* expression in SCA1 BG could thus be a compensatory mechanism to promote formation of new excitatory synapses as these signaling structures are lost. Expression of growth factors, such as Fibroblast growth factor (*Fgf) 12*, and *Platelet Derived Growth Factor D (PdgfD)* was also increased in SCA1 BG. Fibroblast growth factors (FGFs) via their receptors (FGFRs) modulate important cellular processes such as cell proliferation, and death. FGF12 interacts with all four major FGFR and protects cells from apoptosis (Sochacka et al., 2020). In response to injury or various stresses, PDGFs modulate neuronal excitability by affecting ion channels, and stimulate survival signals rescuing cells from apoptosis (Funa and Sasahara, 2014). Increased expression of *Fgf12* and *PdgfD* in BG is likely to be neuroprotective and delay dysfunction and cell death of PCs (Chopra et al., 2018). In addition, BG may be serving a neuroprotective role by upregulating the expression of the *Cystatin 3* (*Cst3*), the most abundant extracellular inhibitor of cysteine proteases. Cysteine proteases are upregulated in neurodegenerative and neuroinflammatory conditions and can lead to ECM breakdown and cell death (Hook et al., 2015). Inhibitors of cysteine proteases including Cst3 have been shown to be neuroprotective in neurodegenerative diseases (Kaur and Levy, 2012; Zou et al., 2017). Thus, increase in *Cst3* in BG could be neuroprotective by preventing deleterious ECM changes. Together, increased expression of these neuro-supportive genes indicates potential mechanisms by which reactive SCA1 BG exert beneficial effects during the early stages of disease.

We have also observed changes in the expression of homeostatic BG genes responsible for regulating neuronal function. We found that expression of *Kcnj10* (Figure 3D), encoding the potassium inward rectifier Kir4.1, is decreased in BG at this stage of disease. Kir4.1 regulates extracellular potassium ion levels and, subsequently, PC excitability and firing rate. Previous work has shown that the firing rate of PCs is decreased early in SCA1 (Dell’Orco et al., 2015). This observed decrease in *Kcnj10* expression is expected to moderate PC firing rate (Djukic et al., 2007). BG may also moderate PC synaptic signaling via reduced expression of *Solute carrier family 1 Member 3 (Slc1a3). Slc1a3* encodes for the plasma membrane glutamate aspartate transporter (GLAST) that is responsible for synaptic glutamate removal. Decreased *Slc1a3* expression may result in slower and less efficient glutamate removal, thus prolonging glutamate signaling on PCs.

Our results also provide insight into how are BG genes altered in SCA1. Previous studies have found that PCs regulate BG character via Sonic hedgehog (Shh) signaling (Farmer et al., 2016). For instance, Shh signaling from PCs regulates higher expression of *Kcnj10* and lower expression of *aquaporin 4* (*Aqp4)* in BG compared to velate astrocytes. Interestingly, we found that expression of Shh receptors *Patched 1* and *2* (*Ptch1, Ptch2*) (Adolphe et al., 2014) is decreased in BG in *Pcp2-ATXN1[82Q]* cerebella indicating one critical way in which PC-BG communication is impacted in SCA1. Loss of PC-BG Shh signaling could affect BG function in several ways, including decreased expression of *Kcnj10* and increased expression of *Aqp4*, both changes that we indeed observed in SCA1 BG. Expression of Shh downstream transcriptional activator *Gli1* is also decreased, further supporting dysfunctional Shh PC-BG signaling in SCA1 (Figure 3D). We investigated whether these changes in Shh signaling occur widely or only in the subset of BG, such as the ones residing next to *hATXN1*+ PCs. Most BG observed in this study showed decreased expression of *Ptch1, Ptch2*, and *Gli1* (Supplementary Figure 6A). We confirmed reduced expression of *Ptch2* and *Gli1* using RT-qPCR of whole *Pcp2-ATXN1[82Q]* cerebellar extracts (Supplementary Figure 6B and Supplementary Table 1). As the most logical cause of this reduced BG expression is decreased expression of ligand *Shh* from PCs, we assessed *Shh* expression in identified PC populations. However, we found no significant change in *Shh* expression in total PCs, *hATXN1*+, or *hATXN1*- PCs (Supplementary Figure 6C and Supplementary Table 5). This may suggest either altered post-transcriptional regulation of Shh in PCs or broader changes in PC signaling capabilities that are disrupting this crucial mechanism.

We further investigated the expression of genes that may regulate BG response to PC dysfunction. Calcium regulated adenylate cyclase *(Adcy8)* controls neuroinflammation by increasing the production of cyclic adenosine monophosphate (cAMP), an important negative regulator of inflammation (Wieczorek et al., 2012; Wang et al., 2020). We have found increased expression of *Adcy8* in BG that may moderate their pro-inflammatory response in SCA1. Sox2 is a transcriptional regulator of BG reactivity. The long non-coding RNA (lncRNA) *Rmst* is known for its role in facilitating activation of Sox2. We observed increased expression of *Rmst* that may play a role in facilitating Sox2-regulation of reactive glial response in SCA1 BG (Ng et al., 2013; Cerrato et al., 2018).

Hierarchical enriched pathways analysis of the DEGs in BG identified pathways involved in development, including cell junction assembly, positive regulation of cell projection organization, regulation of neurogenesis, axonogenesis, gliogenesis, small molecule metabolic processes and neuron apoptotic processes (Figure 3E). Among top GO pathways associated with the upregulated genes were nervous system development (*q* = 1.2 × 10^–9^), regulation of biological quality (*q* = 1.5 × 10^–9^), cell adhesion (q = 9.8 × 10^–9^), organonitrogen compound metabolic processes (*q* = 3.8 × 10^–7^) and cell projection organization (*q* = 5 × 10^–8^). KEGG identified circadian entrainment (*q* = 3.6 × 10^–2^) as dysregulated pathway in BG. This is very intriguing considering the role the cerebellum has in sleep and sleep disturbances in SCA patients (Pedroso et al., 2011; DelRosso and Hoque, 2014). GO pathway analysis of downregulated genes identified transmembrane transporter binding (*q* = 7 × 10^–3^), smoothened binding and hedgehog receptor activity (*q* = 4.9 × 10^–2^), cell to cell signaling (*q* = 6.4 × 10^–7^), apoptotic processes (*q* = 1.3 × 10^–2^) and synapse (*q* = 5 × 10^–14^). KEGG analysis of downregulated genes identified retrograde endocannabinoid signaling (*q* = 1 × 10^–3^), glutamatergic (*q* = 2 × 10^–3^) and dopaminergic synapses (*q* = 4.3 × 10^–2^), long-term depression (*q* = 8.1 × 10^–3^) and Spinocerebellar ataxias (*q* = 8.4 × 10^–3^).

Our analysis supports the hypothesis that SCA1 BG increase their neuroprotective support in response to PC dysfunction. It confirms the reactive SCA1 phenotype previously observed through the upregulation of *Gfap* and suggests an ongoing regulation of neuroinflammatory response through the expression of *Adcy8* and *Rmst.* More importantly, these results implicate altered PC-BG Shh signaling as one of the mechanisms by which BG function is perturbed in SCA1.

### Increased expression of genes that promote neurogenesis, gliogenesis, and synaptogenesis indicates compensatory gene expression changes in velate astrocytes

Velate astrocytes (VAs) are a type of cerebellar astrocytes that reside in the granule cell layer. As such, they are surrounded by the most numerous neurons in the brain and are the only astrocytes in the brain that are largely outnumbered by neurons they are associated with (Herculano-Houzel, 2014). However, very little is known about the response of velate astrocytes to cerebellar pathology, including SCA1. Therefore, we next investigated genes and pathways altered in SCA1 velate astrocytes.

**FIGURE 4:**
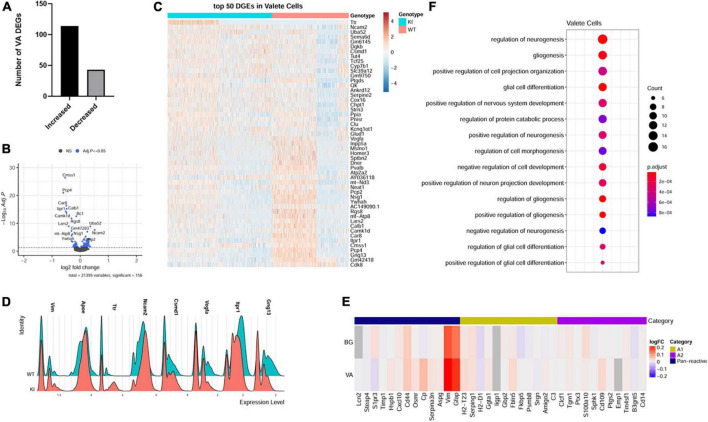
Velate astrocytes gene expression changes in SCA1. For identified velate astrocytes limma was used to test differential gene abundance between control and *Pcp2-ATXN1[82Q]* samples. *N* = 3 mice of each genotype. *P* values were adjusted using Benjamini-Hockberg method. Differential gene expression was determined by an adjusted *p*-values of 0.05. **(A)** Number of upregulated and downregulated differentially expressed genes (DEGs) in VA. **(B)** Volcano plot showing DEGs of VA cluster in *Pcp2-ATXN1[82Q]* SCA1. **(C)** Heatplot displaying expression profiles of top 25 highest upregulated and downregulated DEGs in wild-type and *Pcp2-ATXN1[82Q]* VA determined by logFC values with adjusted p-values ≤ 0.05. *N* = 3 mice of each genotype. **(D)** Ridgeplot showing distribution of expression of selected genes in wild-type and *Pcp2-ATXN1[82Q]* VAs. **(E)** Heatplot of normalized expression of astrocyte A1/A2 genes in BG and VA at 12 weeks of age. Color indicates logFC. **(F)** Dot plot of the pathway analysis of total DEGs between WT and *Pcp2-ATXN1[82Q]* VA at 12 weeks of age. The color of dots depicts the adjusted p values, radius of the dot depicts gene counts (number of genes in the enriched pathway).

We have identified 157 DEGs (p.adj < = 0.05) in SCA1 velate astrocytes compared to those of their wild-type littermate controls. A majority (114 or 72.6%) of the identified DEGs were upregulated (Figures 4A–D). Notably, one of genes with increased expression was *Vimentin* (*Vim*). *Vim* encodes for the intermediate filament predominantly found in astrocytes. Vimentin is upregulated in reactive astrogliosis (Janeczko, 1993; O’Leary et al., 2020), indicating that VAs, like BG, undergo reactive gliosis in SCA1 (Figure 4D). *Apolipoprotein E* (*ApoE*) also plays a role in reactive astrogliosis (Ophir et al., 2003; Sen et al., 2017). Increased expression of *ApoE* in VAs further indicates reactive velate astrogliosis in SCA1.

A previous study identified two different types of reactive astrocytes that were termed “A1” and “A2,” respectively (Liddelow et al., 2017). A1 astrocytes are thought to be harmful as they up-regulate classical complement cascade genes previously shown to be destructive to synapses, while A2 astrocytes are thought to be neuroprotective due to increased expression of neurotrophic factors. To determine whether reactive BG and VAs are more like A1 or A2 astrocytes, we examined the expression of A1 and A2 astrocyte genes in VA and BG. While we found increased expression of panreactive genes *Gfap*, *Vim*, and *CD44*, we found no clear pattern or distinction in A1 or A2 type gene expression in BG or VAs (Figure 4E).

Among the top upregulated VA genes were genes involved in neurogenesis, gliogenesis, and synapse maintenance including *Neural Cell adhesion molecule 2* (*Ncam2*), *Sema6D*, *Quaking (Qk)*, *Serpine E2*, *Clusterin (Clu)* and *CUB And Sushi Multiple Domains 1* (*Csmd1*). NCAM2 is involved in many roles in the brain including neurogenesis, neuronal migration, neuronal differentiation, synaptogenesis, calcium signaling, and maintenance of presynaptic and postsynaptic compartments in adult brains. In addition, it has been proposed that a decrease in NCAM2 levels is associated with loss of synaptic structure in the early stages of neurodegenerative diseases (Parcerisas et al., 2021). Quaking (Qk) is an RNA binding protein residing in astrocyte processes and promotes astrocyte maturation (Sakers et al., 2021). Astrocyte secreted Clu co-localizes with presynaptic puncta of excitatory neurons and loss of *Clu* led to impaired presynaptic function and reduced spine density *in vivo* (Chen et al., 2021). On the other hand, overexpression of *Clu* in astrocytes reduced pathology and restored synaptic function in mouse model of Alzheimer’s disease (Chen et al., 2021). Recently, *CUB And Sushi Multiple Domains 1*(*Csmd1*) was suggested to oppose the complement cascade that facilitates synaptic loss in neurodegeneration (Baum et al., 2020). Therefore, it is reasonable to assume that increased expression of *Ncam2*, *Qk*, *Clu* and *Csmd1* in velate astrocytes may be neuroprotective in SCA1 by promoting astrocyte maturation and synaptic function.

VEGF is a neurotrophic factor whose decrease was previously shown to contribute to Purkinje cell pathology in SCA1 (Cvetanovic et al., 2011). We found a reduced expression of *vascular endothelial growt*h *factor* (*VEGF)* suggesting that velate astrocytes contribute to a VEGF reduction in SCA1 (Figure 4D).

Pathway analysis of all VA DEGs identified developmental pathways including regulation of neurogenesis, gliogenesis, positive regulation of projection organization, regulation of nervous system development, and regulation of protein catabolic process (Figure 4F). Breaking this down into pathways unique to up and downregulated genes, we found that GO pathway analysis of only upregulated genes highlighted many developmental pathways including cell morphogenesis, regulation of neuron projection development, positive regulation of gliogenesis, and glial differentiation (*q* values of 3.2 × 10^–6^, 1.5 × 10^–5^, 3.9 × 10^–5^, and 4.5 × 10^–5^, respectively). Further, GO pathway analysis of the downregulated genes identified calcium ion binding (*q* = 3.1 × 10^–3^), vesicle mediated transport in synapse (*q* = 3.5 × 10^–2^), negative regulation of neuronal death (*q* = 4.1 × 10^–2^), glutamatergic synapse (*q* = 4.5 × 10^–4^), Spinocerebellar ataxia (*q* = 1.3 × 10^–3^), retrograde endocannabinoid signaling (*q* = 2.4 × 10^–2^), and long-term depression (*q* = 2.7 × 10^–2^).

Together, these results indicate that VAs exhibit a reactive astrocyte phenotype in SCA1 that is characterized by the expression of genes regulating synapse structure and maintenance. Furthermore, reduction in *Vegf* expression in VAs seems to implicate VAs as an important contributor to reduced VEGF expression in the SCA1 cerebellum, suggesting that they may play a role in PC pathology. As this model represents a non-cell autonomous response of these cells to PC dysfunction this could indicate a negative feedback mechanism that could contribute to progressive worsening of cellular pathology over time.

### Gene expression analysis indicates reactive activation in Spinocerebellar ataxia type 1 oligodendrocytes

Oligodendrocytes (OL) envelop axons with myelin and maintain long-term axonal integrity (Bradl and Lassmann, 2010). Cells of the oligodendrocyte lineage are important for cerebellar development and motor learning (Mathis et al., 2003; McKenzie et al., 2014). Previous studies have found significant changes in the cerebellar white matter in SCA1 patients and in *Pcp2-ATXN1[82Q]* mice, indicating that oligodendrocytes (OLs) may be affected in SCA1 (Liu et al., 2018; Park et al., 2020). Moreover, a recent study found a decrease in oligodendrocyte numbers in human post-mortem samples from patients with SCA1 (Tejwani et al., 2021) and a decreased expression of mature oligodendrocyte genes, including *Myelin Oligodendrocyte Glycoprotein* (*Mog*) in a knock-in mouse model of SCA1, *Atxn1^154^Q*/2*^Q^* mice (Tejwani et al., 2021). In *Atxn1*^154^*Q*/2*^Q^* mice mutant Atxn1 is expressed widely, including in oligodendrocyte precursor cells (OPC) and OLs.

**FIGURE 5:**
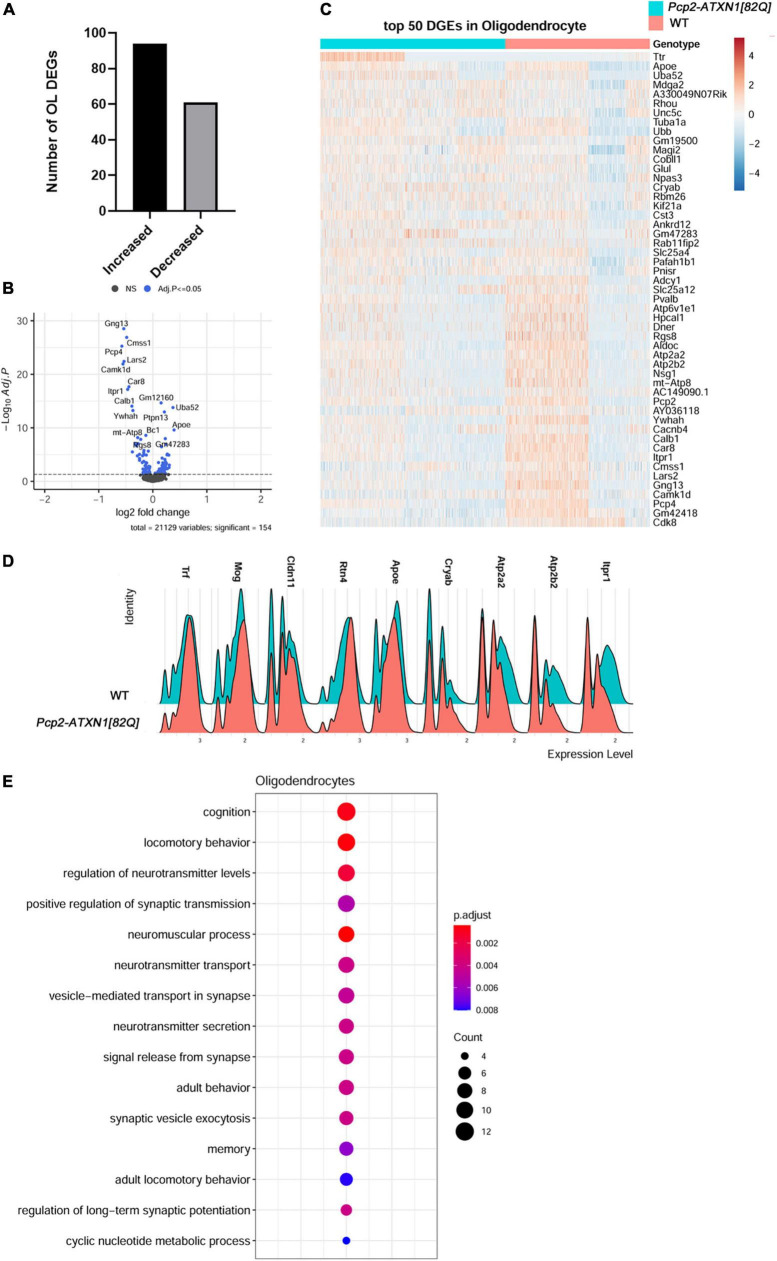
Gene expression changes in SCA1 oligodendrocytes. Limma was used to test differential gene abundance in oligodendrocytes between control and *Pcp2-ATXN1[82Q]* samples. *N* = 3 mice of each genotype. *P* values were adjusted using Benjamini-Hockberg method. Differential gene expression was determined by an adjusted *p*-values of 0.05. **(A)** Number of upregulated and downregulated differentially expressed genes (DEGs) in OL. **(B)** Volcano plot showing DEGs of OL cluster in *Pcp2-ATXN1[82Q]* SCA1. **(C)** Heatplot displaying expression profiles of top 25 highest upregulated and downregulated DEGs in wild-type and *Pcp2-ATXN1[82Q]* oligodendrocytes determined by logFC values with adjusted *p*-values ≤0.05. *N* = 3 mice of each genotype. **(D)** Ridgeplot showing distribution of expression of selected genes in wild-type and *Pcp2-ATXN1[82Q]* OL. **(E)** Dot plot of the pathway analysis of total DEGs between WT and *Pcp2-ATXN1[82Q]* OL at 12 weeks of age. The color of dots depicts the adjusted p values, radius of the dot depicts gene counts (number of genes in the enriched pathway).

To gain insight into which of these gene expression changes are induced in response to PC pathogenesis alone, we examined genes and pathways altered in oligodendrocyte nuclei from *Pcp2-ATXN1[82Q]* mice compared to littermate controls. We found 154 DEGs in oligodendrocytes in *Pcp2*-*ATXN1[82Q]* cerebella, with the majority (94 DEG or 61%) of these genes being upregulated (Figure 5A). While the percentage of OLs identified in our SCA1 samples is not significantly different from WT controls, the expression of several oligodendrocyte marker genes including *Transferrin* (*Trf)*, *Mog*, *Claudin 11* (*Cldn11*) and *Reticulon 4* (*Rtn4*) was increased (Figures 5B–D; Bradl and Lassmann, 2010).

Increased expressions of *Crystallin Alpha B* (*Cryab)* and *apolipoprotein E* (*ApoE)* have been associated with protective reactive gliosis in neurodegenerative diseases such as Parkinson’s disease (PD), Alzheimer’s disease (AD), and Multiple Sclerosis (MS) (Ophir et al., 2003; Ousman et al., 2007; Liu et al., 2015; Kuipers et al., 2017; D’Agostino et al., 2019). We found increased expression of *Cryab* and *ApoE* indicating protective reactive oligodendrogliosis in SCA1.Notably, we observed increased expression of transcriptional factor *JunD*, which is a functional component of activated protein 1 (AP1) complex. The AP1 complex regulates many OL inflammatory and immune genes, suggesting that AP1 may modulate a non-cell autonomous reactive OL response in SCA1 pathogenesis (Behmoaras et al., 2008).

We also found altered expression of many genes important for oligodendrocyte function (Figure 5E). Among them is increased expression of *Glutamate-Ammonia Ligase* (*Glul*) encoding for glutamine synthetase (GS) This molecule serves as a key glutamate-catabolizing enzyme that catalyzes the conversion of glutamate to glutamine. Previous studies demonstrated that OLs support neuronal glutamatergic transmission via expression of GS (Xin et al., 2019). Moreover, *Glul* expression in OLs is increased in chronic pathological conditions including amyotrophic lateral sclerosis (ALS) and MS (Ben Haim et al., 2021). As glutamate signaling is perturbed in SCA1, it is possible that OLs increase GS expression to compensate for these changes in glutamate metabolism in SCA1.

Genes downregulated in SCA1 OLs included *Atp2a2* and *Atp2a3* which encode for the calcium endoplasmic reticulum pumps SERCA1 and SERCA2, respectively. This implicates a reduced ability of OLs endoplasmic reticula (ER) to buffer calcium. In addition, reduced expression of *Atp2b2* that encodes for Plasma Membrane Calcium-Transporting ATPase is expected to reduce plasma membrane calcium buffering. Together these results indicate perturbed calcium homeostasis as a feature of reactive SCA1 OLs (Figures 5B-D).

Intriguingly, the top two pathways of OLs DEGs identified were cognition and locomotor behavior, implicating that molecular pathway alterations in SCA1 oligodendrocytes could be associated with the broader symptomology of SCA1. Pathway analysis of all OLs DEGs also identified regulation of neurotransmitter levels, positive regulation of synaptic transmission, vesicle-mediated transport in synapses and neurotransmitter secretion, potentially implicating OL in modulating synaptic transmission in SCA1 (Figure 5E).

Breaking this analysis down into pathways unique to up and downregulated genes, we found that GO pathway analysis of only downregulated genes identified pathways involved in transport and binding of ions including inorganic cation transmembrane transporter activity (q = 7.5 × 10^–6^), calcium binding (q = 9.3 × 10^–6^), P-type calcium transporter activity (q = 4.2 × 10^–4^), synaptic signaling (q = 1.6 × 10^–7^), modulation of chemical synaptic transmission (q = 5.4 × 10^–6^), glutamatergic synapse (q = 6.4 × 10^–6^), and Spinocerebellar ataxia (q = 2.9 × 10^–5^). Moreover, pathway analysis of OLs upregulated DEGs identified movement of cell or subcellular component (q = 9.4 × 10^–5^), cell migration (q = 8.8 × 10^–4^), myelin sheath (q = 2.4 × 10^–7^), and synapse (q = 1.4 × 10^–4^).

Together, these analyses indicate that oligodendrocytes respond to PC dysfunction in SCA1 by becoming reactive. Reactive response of SCA1 OLs is characterized by increased expression of *Cryab*, *ApoE, and Glul*, and may be regulated by transcriptional factor AP1. While this may allow for increased neuroprotection, such as compensatory glutamate buffering, reactive OLs response may impact their calcium homeostasis.

## Discussion

Through this work we sought to increase our understanding of the cell-type specific changes during the early stages of SCA1. Furthermore, by using a model in which the pathological mutation was restricted to PCs, we aimed to provide insight into how these pathogenic processes within PCs affect non-cell autonomous gene expression and signaling pathways within other cerebellar cells. To accomplish this, we have employed single nuclei RNA sequencing to investigate transcriptional changes in individual cerebellar cell types in “early stage” 12 week old animals, a time point which represents a phase of SCA1 in which symptoms are beginning to become apparent but before the progressive cell death of PCs within the cerebellum.

There are several important findings from our study. First, mutant ATXN1 expression in Purkinje cells causes profound transcriptional alterations in cerebellar glia in a non-cell autonomous manner. Mutant ATXN1 expression in PCs alone caused altered gene expression in every glial subtype analyzed, often driving an increase in gene expression (Bergmann glia (62.8%), velate astrocytes (72.6%) and oligodendrocytes (61%). As at this early disease stage there is no detectable Purkinje cell loss in *Pcp2-ATXN1[82Q]* mice, we propose that these glial transcriptome changes are in direct response to ATXN1-driven Purkinje cell dysfunction (Bell et al., 2010; Ingram et al., 2016b).

Second, we identified novel transcriptional changes in PCs that may be relevant to disease pathogenesis. These potentially compensatory gene expression changes include increased expression of *Ralyl*, *Atp6v0c*, *Atpgv0a1*, *Atp6v1h*, Atp6v and *Slc32a1. Ralyl* is an RNA binding protein known for its neuroprotective role in Alzheimer’s (Zhang et al., 2020). Increased expression of three vacuolar V-type ATP-ase subunits (*Atp6v0c*, *Atpgv0a1*, and *Atp6v1h*) that provide electrochemical gradient to fill synaptic vesicles with GABA, and *Slc32a1*, encoding vesicular GABA and Glycine Transporter (VGAT) that transports GABA in the vesicles may suggest increased GABA content in PCs synaptic vesicle. As spontaneous firing rate of PCs is decreased in *Pcp2-ATXN1[82Q]* mice (Dell’Orco et al., 2015), an increase in GABA loading per vesicle may represent compensatory change to restore or ameliorate synaptic transmission by increasing the total amount of GABA released at PC terminals.

Third, because previous work in this mouse model suggested altered levels of mutant ATXN1 in PCs across the cerebellum, we assessed the relative expression of human ATXN1 within our SCA1 PCs. In doing so, we identified two populations- those that were expressing detectable ATXN1 (∼25%) and those that had no detectable ATXN1. It is likely that we underestimated the number of mutant ATXN1 expressing PCs due to the rigor of our analysis. It is unclear whether PCs with undetectable mutant *hATXN1* have reduced production of mutant ATXN1 mRNA or are more efficient in transporting it out of the nucleus and/or degrading it. Intriguingly, in contrast to prevalent gene downregulation in total PCs and *hATXN1- PCs*, majority of DEGs were upregulated in *hATXN1*+ cells. It is possible that these upregulated genes provide resistance to SCA1, and it will be important for future studies to distinguish whether *hATXN1*+ or – cells are more affected in SCA1. This result also indicates that some of the ATXN1 –induced gene expression changes may be missed when comparing total SCA1 to WT PCs. Furthermore, out of 370 DEGs in *hATXN1- PCs*, 355 were also present when comparing total SCA1 to WT PCs. Thus majority (64%) of DEGs identified in comparison of total SCA1 and WT PCs are genes with altered expression in PCs in which we could not detect mutant *hATXN1.*

Fourth, our results support perturbed Shh signaling as one of contributors to BG molecular alterations in SCA1. Sonic hedgehog (Shh) signaling is one of the key communication pathways by which PCs regulate BG character (Farmer et al., 2016). We found that the expression of Shh receptors *Patched 1* and 2 and Shh signaling downstream transcription factor *Gli1* are reduced in SCA1 BG. Previous studies have shown that Shh-Ptch2 communication regulates expression of homeostatic genes *Slc1a3*, *Kcnj10*, and *Aqp4* in Bergmann glia (Farmer et al., 2016). We also validated a previously reported decrease in the expression of glutamate transporter *Slc1a3* in Bergmann glia (Cvetanovic, 2015), and identified additional perturbations in the expression of homeostatic genes such as *Kcnj10 and Aqp4* that are critical for PC function (Djukic et al., 2007; Nicita et al., 2018). Therefore, we propose that decreased Shh signaling contributes to reduced expression of S*lc1a3* and *Kcnj10* and increased expression of *Aqp4* that we found in SCA1 BG.

Fifth, we provide evidence that reactive response in velate astrocytes includes increased expression of several neuroprotective genes such as *ApoE, Ncam2*, *Clu* and *Csmd1*. ApoE, among other roles, contributes to astrocyte activation and increases BDNF secretion. Increased ApoE in SCA1 VAs may contribute to increased BDNF that is neuroprotective in SCA1 (Ophir et al., 2003; Sen et al., 2017; Mellesmoen et al., 2018; Sheeler et al., 2021). *Ncam2, Clu* and *Csmd1* are protective against loss of synapses (Baum et al., 2020; Parcerisas et al., 2021), and their increased expression in SCA1 VAs may compensate for loss of synapses seen in SCA1. These results also increase our limited understanding of VAs reactive gliosis in cerebellar disease.

Six, we identified intriguing transcriptional changes in SCA1 oligodendrocytes indicative of reactive oligodendrogliosis that may be regulated by increased expression of JunD. While OLs numbers do not seem to be altered at 12 weeks in *Pcp2-ATXN1[82Q]* cerebellum, we have found increased expression of OL marker genes and neurosupportive genes including *Trf*, *Mog*, *Cldn11*, *Rtn4*, *Glul, Cryab* and *ApoE*. These results indicate neuroprotective effects of reactive cerebellar OLs in SCA1.

Finally, we identified shared DEGs and pathways that are altered in all four analyzed cell types in SCA1 cerebella and as such are promising candidates for future therapies. Transthyretin (Ttr) is a protein that binds and distributes retinol and thyroid hormones. Retinol is important for cerebellar function (Tafti and Ghyselinck, 2007) where it binds retinoic acid receptor-related orphan receptors (ROR). Previous studies identified the importance of retinol in SCA1 (Serra et al., 2006), by demonstrating reduced expression of RORa–regulated genes in cerebella of SCA1 mice, and that partial loss of RORa enhances PCs pathology (Serra et al., 2006). Recent studies found that Ttr plays important roles in other cells such as OPC, cerebellar granule neurons and astrocytes. For example, Ttr regulates proliferation and survival of OPCs (Alshehri et al., 2020), δ-GABAA-R expression in cerebellar granule neurons (Zhou et al., 2019), and glycolysis in astrocytes (Zawişlak et al., 2017). We found that *Ttr* expression is increased in SCA1 PCs, BG, VA and OL. It is likely that this increase in *Ttr* expression is beneficial for PCs, OLs, astrocytes and granule neurons, and compensates for reduced RORa function in SCA1. It will be important to understand the mechanism of *Ttr* upregulation, and whether exogenous Ttr is therapeutically beneficial in SCA1.

In addition, several Magenta module genes, including genes involved in calcium homeostasis, such as *Atp2a2* and *Itpr1* were reduced in all four cell types, indicating that restoring calcium homeostasis may be another good target for SCA1 therapeutic approaches.

Selective neuronal vulnerability is a feature shared by many neurodegenerative diseases. In the case of SCA1, although mutant ATXN1 is expressed throughout the brain, cerebellar Purkinje cells (PCs) are most affected (Servadio et al., 1995). Severe vulnerability of PCs in SCA1 is likely brought about by the combination of the toxic effects of mutant ATXN1 within PCs and the changes in PCs microenvironment, including glial cell alterations. In this respect, it is important to note that glial cells in the cerebellum are found to have distinct transcriptomes compared to the glial cells in other brain regions (Grabert et al., 2016; Soreq et al., 2017; Boisvert et al., 2018). Uniqueness of cerebellar glia gets even more pronounced during aging (Grabert et al., 2016; Boisvert et al., 2018; Clarke et al., 2018). Yet, while neurodegeneration associated glial gene expression changes have been studied intensively in the other brain regions, less is known about gene expression changes in cerebellar glia during neurodegeneration. Our results provide much needed insight into gene expression changes of cerebellar Bergmann glia, velate astrocytes and oligodendrocytes in response to PC dysfunction.

It is likely that some of the molecular changes we identified are compensatory, allowing for continued cerebellar function, and that some may be pathogenic, promoting disease progression.

Our results provide a framework to investigate how individual pathogenic processes contribute to the sequence of progressive cerebellar dysfunctions in SCA1. Our results are freely available and we hope that they will stimulate future studies to causally investigate how these gene and pathway perturbations contribute to SCA1 pathogenesis as well as facilitate development of novel therapeutic approaches.

## Data availability statement

All the data from this study are available from the authors. RNA sequencing data are deposited at https://www.ncbi.nlm.nih.gov/geo/query/acc.cgi?acc=GSE215336.

## Ethics statement

Animal experimentation was approved by Institutional Animal Care and Use Committee (IACUC) of University of Minnesota.

## Author contributions

MC conceptualized the study. EB, CS, KH, FM, and KS performed the experiments. YZ and MC analyzed the data. MC, EB, CS, KH, FM, KS, and YZ wrote the manuscript. All authors contributed to the article and approved the submitted version.

## Supplementary material

Supplementary Figure 1 Example of isolated nuclei showing absence of clumping and spherical, non-damaged nuclei. Nuclei for RNA sequencing were isolated from the mouse cerebella using detergent mechanical lysis protocol, and stained with DAPI. Scale bar = 100 μm.

Supplementary Figure 2 Reduced expression of Purkinje cell genes in *Pcp2-ATXN1[82Q]* mice. Quantitative RT-PCR of bulk cerebellar lysates was used to evaluate expression of Purkinje cell genes *Calb1*, *Pcp4*, *Homer3*, *Rgs8*, *ITPR*, *Inpp5* and *Garnl3.* Data is presented as mean ± SEM with average values for each mouse represented by a dot. *N* = 6 -7 mice per genotype (WT and *Pcp2-ATXN1[82Q]*) **p* < 0.05 unpaired t test with Welch’s correction.

Supplementary Figure 3 Expression of endogenous mouse *Atxn1[2Q]* and mutant human *ATXN1[82Q]* in cerebellar cells. **(A)** Percentage of cerebellar cells expressing endogenous mouse *Atxn1[2Q]* in wild-type and *Pcp2-ATXN1[82Q]* mice. **(B)** Percentage of cerebellar cells expressing mutant human *hATXN1[82Q]* in wild-type and *Pcp2-ATXN1[82Q]* mice. Data is presented as mean ± SEM with average values for each mouse represented by a dot. *N* = 3. **P* < 0.05 Two way ANOVA with Tukey’s multiple comparisons tests.

Supplementary Figure 4 Decreased expression of *Dner* mRNA and protein in Purkinje Cells. **(A).** Quantitative RT-PCR of bulk cerebellar lysates from 12 weeks old wild-type and *Pcp2-ATXN1[82Q]* mice was used to evaluate expression of *Dner*. Data is presented as mean ± SEM with average values for each mouse represented by a dot. *N* = 7, **p* < 0.05 unpaired t test with Welch’s correction. **(B,C)** Cerebellar slices from 12 week old wild-type and *Pcp2-ATXN1[82Q]* mice were stained with DNER antibody. **(D)** Confocal images and Image J were used to quantify DNER expression in soma and dendrites of PCs (right). Data is presented as mean ± SEM with values for each mouse analyzed represented by a dot. *N* = 3 wild-type and *N* = 5 *Pcp2-ATXN1[82Q]* mice**p* < 0.05 unpaired t-test with Welch’s correction. Scale bars = 50 μm.

Supplementary Figure 5 Gene expression changes in *hATXN1* + and *hATXN1-* Purkinje cells. For identified *hATXN1* + and *hATXN1-* Purkinje cells limma was used to test differential expression between control and *Pcp2-ATXN1[82Q]* samples. **(A)** Number of upregulated and downregulated differentially expressed genes (DEGs) in *hATXN1* + and *hATXN1- PCs*. *N* = 3 mice of each genotype. *P* values were adjusted using Benjamini-Hockberg method. Differential gene expression was determined by an adjusted *p*-values ≤0.05. **(B)** Heatplot displaying expression profiles of selected upregulated and downregulated DEGs in wild-type PCs and *hATXN1* + and *hATXN1-* PCs from *Pcp2-ATXN1[82Q]* cerebella determined by logFC values with adjusted *p*-values ≤0.05. *N* = 3 mice of each genotype.

Supplementary Figure 6 Changes in Shh signaling in Bergmann glia. **(A)** Ridgeplots showing distribution of expression of *Ptch1, Ptch2*, and *Gli1* in BG population in wild-type and *Pcp2-ATXN1[82Q]* mice (*N* = 3 of each). *P* values adjusted using Benjamini-Hockberg method ≤0.05 for all three genes. **(B)** Quantitative RT-PCR of bulk cerebellar lysates from 12 weeks old wild-type and *Pcp2-ATXN1[82Q]* mice was used to evaluate expression of *Ptch2* and *Gli1 mRNA.* Data is presented as mean ± SEM with average values for each mouse represented by a dot. *N* = 7, **p* < 0.05 unpaired t test with Welch’s correction. **(C)** Ridgeplot showing distribution of *Shh* expression in *hATXN1* + and *hATXN1-* PCs. *P* value adjusted using Benjamini-Hockberg method ≥0.05.

Supplementary Table 1 Raw data from RTqPCR of PC and BG Genes, DNER intensity.

Supplementary Table 2 Percentage of cells per genotype.

Supplementary Table 3 Percentage of Cell Expressing Endogenous *Atxn1[2Q].*

Supplementary Table 4 Percentage of cells expressing mutant human *ATXN1[82Q].*

Supplementary Table 5 DEG in PCs with detectable and not detectable expression of mutant human *ATXN1*.
