# Supplementary figures and images for: Single nuclei RNA sequencing investigation of the Purkinje cell and glial changes in the cerebellum of transgenic Spinocerebellar ataxia type 1 mice
Source: Front Cell Neurosci. 2022 Nov 15;16:998408. doi: 10.3389/fncel.2022.998408 (PMC9706545; doi:10.3389/fncel.2022.998408)

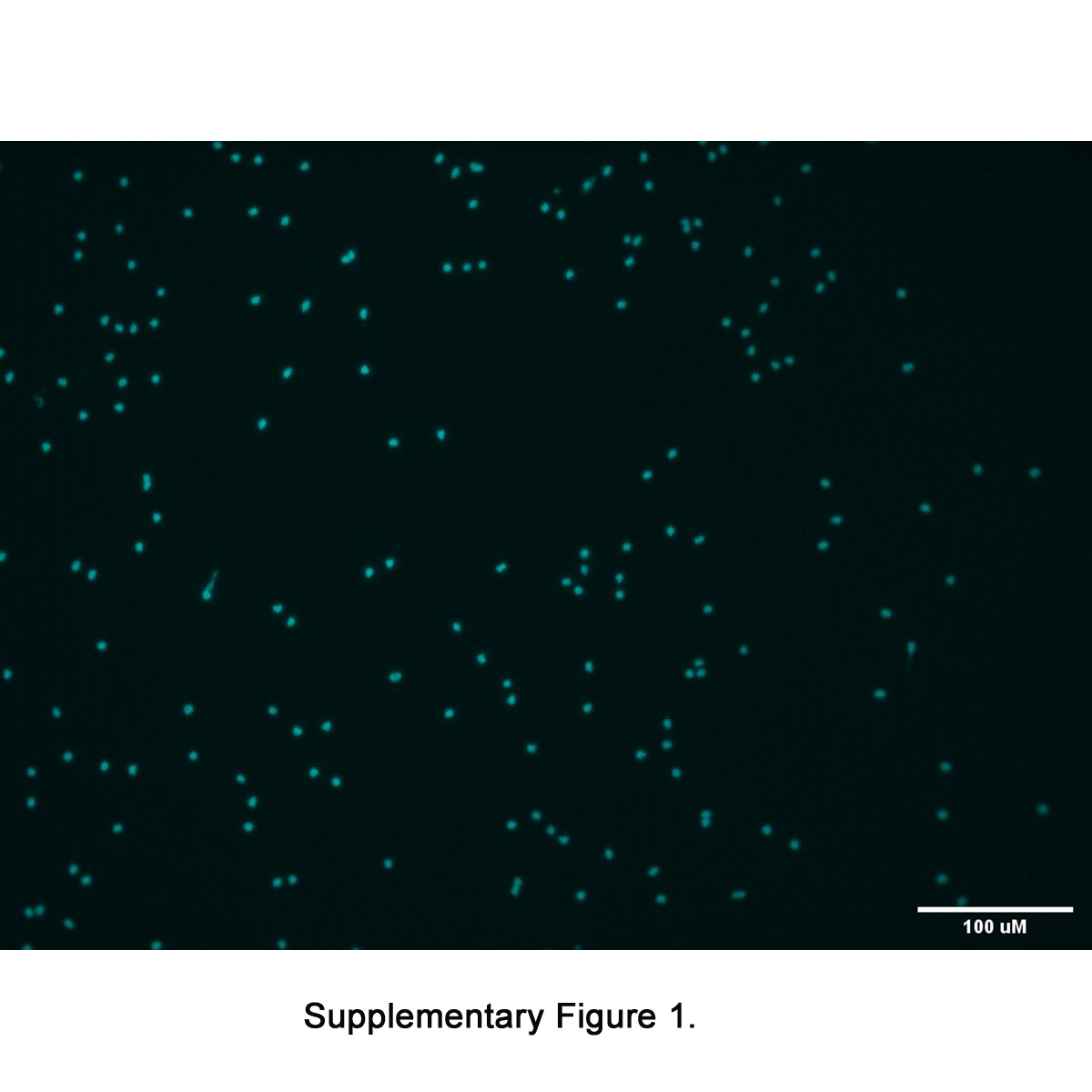

Supplement: Supplementary Figure 1 — Example of isolated nuclei showing absence of clumping and spherical, non-damaged nuclei. Nuclei for RNA sequencing were isolated from the mouse cerebella using detergent mechanical lysis protocol, and stained with DAPI. Scale bar = 100 μm. [file Image_1.TIF]

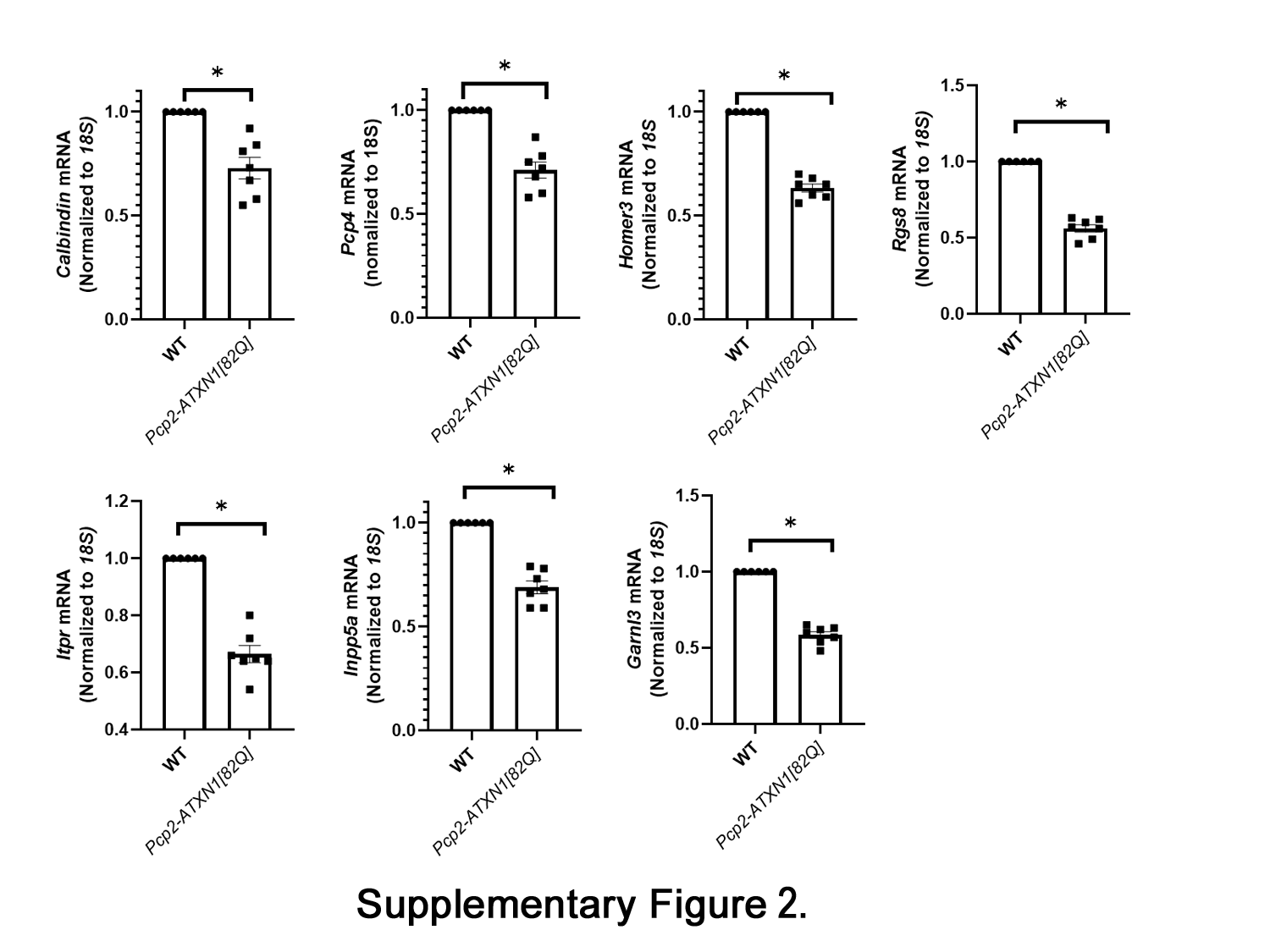

Supplement: Supplementary Figure 2 — Reduced expression of Purkinje cell genes in Pcp2-ATXN1[82Q] mice. Quantitative RT-PCR of bulk cerebellar lysates was used to evaluate expression of Purkinje cell genes Calb1, Pcp4, Homer3, Rgs8, ITPR, Inpp5 and Garnl3. Data is presented as mean ± SEM with average values for each mouse represented by a dot. N = 6 -7 mice per genotype (WT and Pcp2-ATXN1[82Q]) *p < 0.05 unpaired t test with Welch’s correction. [file Image_2.TIF]

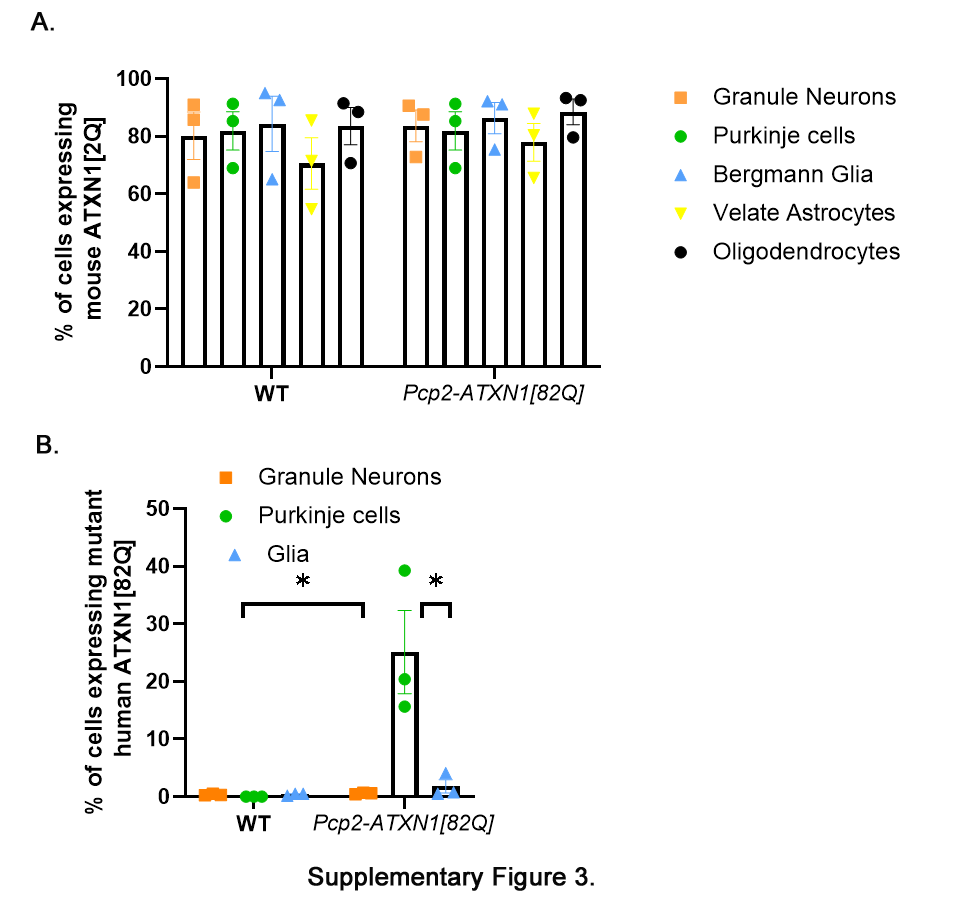

Supplement: Supplementary Figure 3 — Expression of endogenous mouse Atxn1[2Q] and mutant human ATXN1[82Q] in cerebellar cells. (A) Percentage of cerebellar cells expressing endogenous mouse Atxn1[2Q] in wild-type and Pcp2-ATXN1[82Q] mice. (B) Percentage of cerebellar cells expressing mutant human hATXN1[82Q] in wild-type and Pcp2-ATXN1[82Q] mice. Data is presented as mean ± SEM with average values for each mouse represented by a dot. N = 3. *P < 0.05 Two way ANOVA with Tukey’s multiple comparisons tests. [file Image_3.TIF]

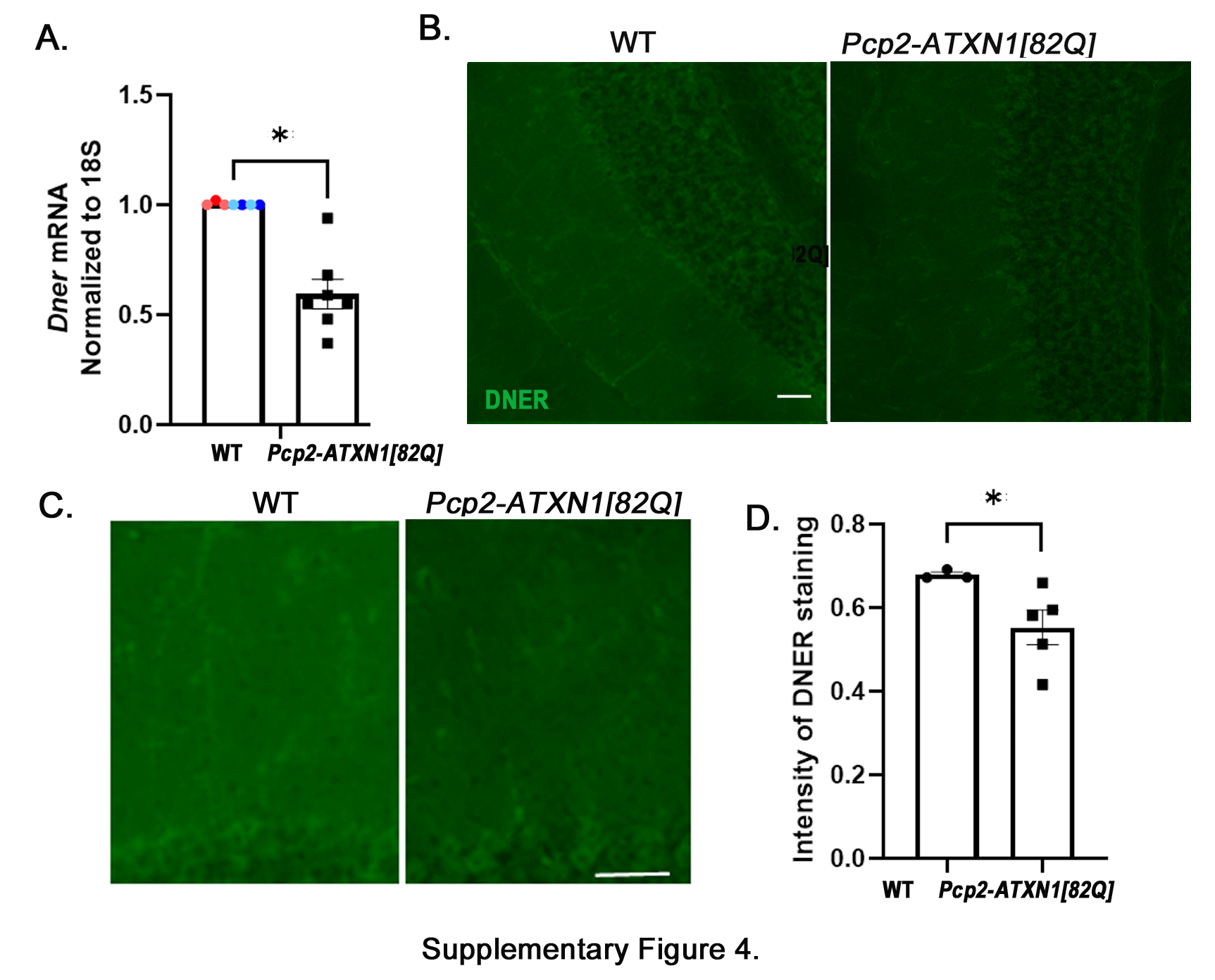

Supplement: Supplementary Figure 4 — Decreased expression of Dner mRNA and protein in Purkinje Cells. (A). Quantitative RT-PCR of bulk cerebellar lysates from 12 weeks old wild-type and Pcp2-ATXN1[82Q] mice was used to evaluate expression of Dner. Data is presented as mean ± SEM with average values for each mouse represented by a dot. N = 7, *p < 0.05 unpaired t test with Welch’s correction. (B,C) Cerebellar slices from 12 week old wild-type and Pcp2-ATXN1[82Q] mice were stained with DNER antibody. (D) Confocal images and Image J were used to quantify DNER expression in soma and dendrites of PCs (right). Data is presented as mean ± SEM with values for each mouse analyzed represented by a dot. N = 3 wild-type and N = 5 Pcp2-ATXN1[82Q] mice*p < 0.05 unpaired t-test with Welch’s correction. Scale bars = 50 μm. [file Image_4.TIF]

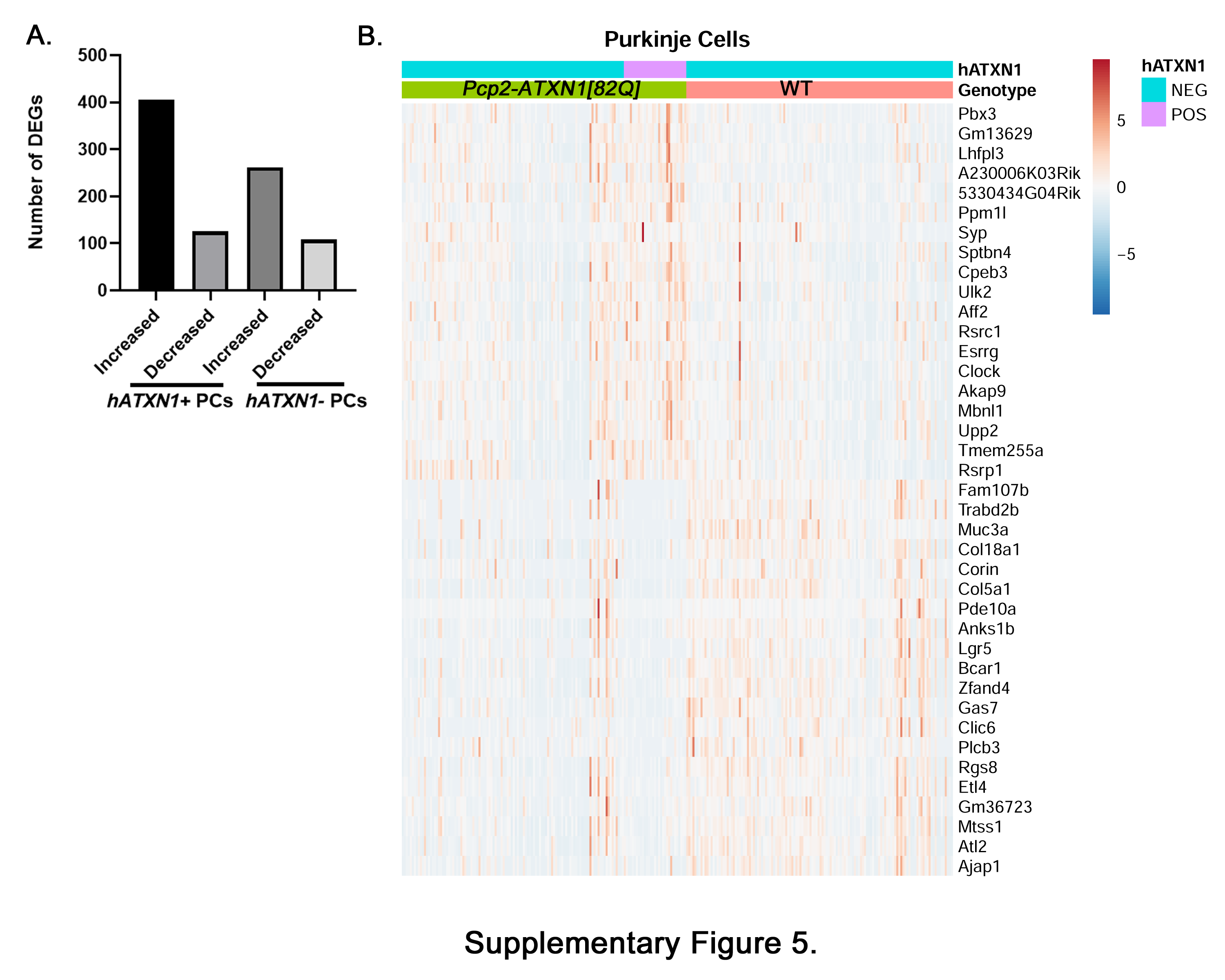

Supplement: Supplementary Figure 5 — Gene expression changes in hATXN1 + and hATXN1- Purkinje cells. For identified hATXN1 + and hATXN1- Purkinje cells limma was used to test differential expression between control and Pcp2-ATXN1[82Q] samples. (A) Number of upregulated and downregulated differentially expressed genes (DEGs) in hATXN1 + and hATXN1- PCs. N = 3 mice of each genotype. P values were adjusted using Benjamini-Hockberg method. Differential gene expression was determined by an adjusted p-values ≤0.05. (B) Heatplot displaying expression profiles of selected upregulated and downregulated DEGs in wild-type PCs and hATXN1 + and hATXN1- PCs from Pcp2-ATXN1[82Q] cerebella determined by logFC values with adjusted p-values ≤0.05. N = 3 mice of each genotype. [file Image_5.TIF]

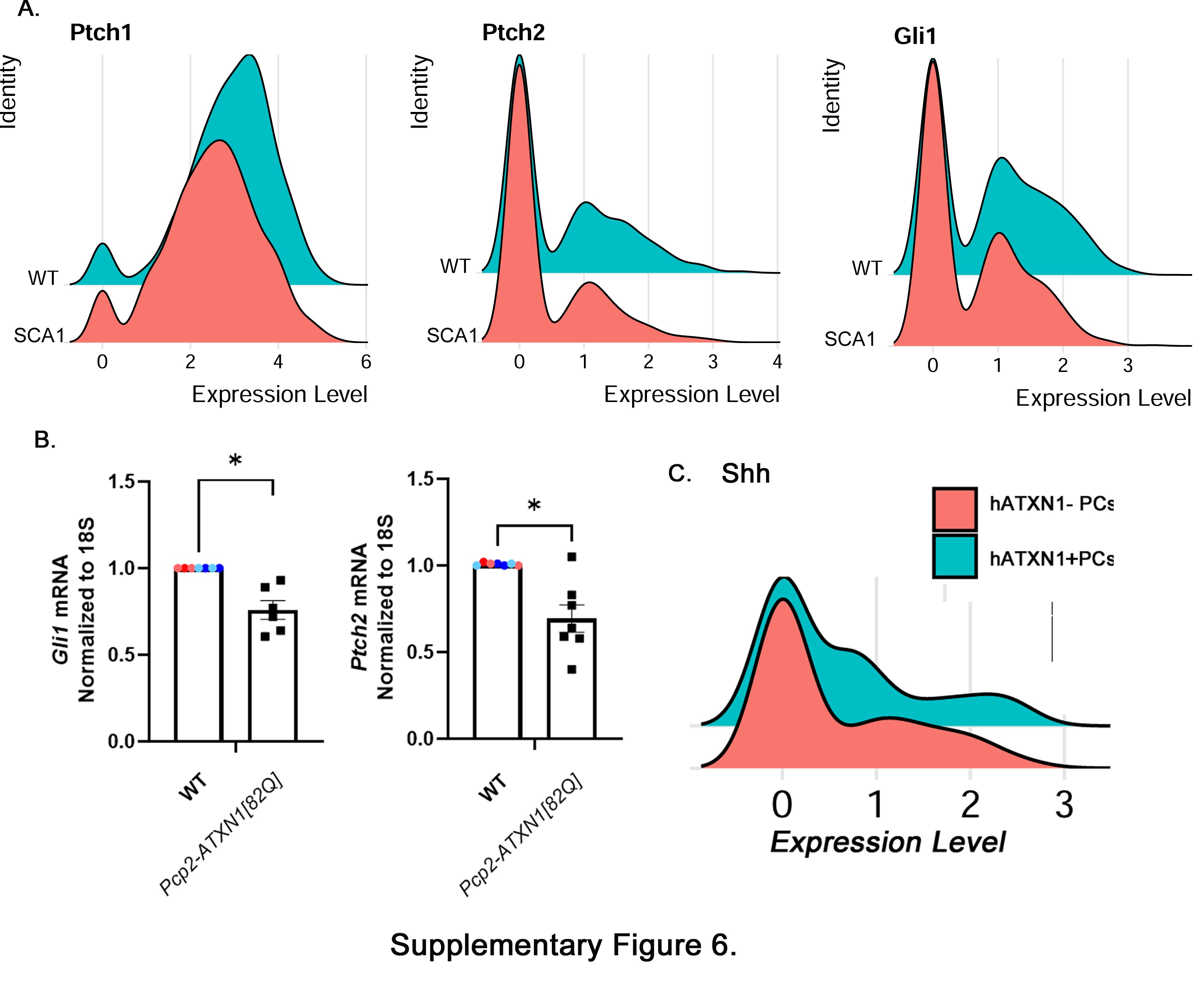

Supplement: Supplementary Figure 6 — Changes in Shh signaling in Bergmann glia. (A) Ridgeplots showing distribution of expression of Ptch1, Ptch2, and Gli1 in BG population in wild-type and Pcp2-ATXN1[82Q] mice (N = 3 of each). P values adjusted using Benjamini-Hockberg method ≤0.05 for all three genes. (B) Quantitative RT-PCR of bulk cerebellar lysates from 12 weeks old wild-type and Pcp2-ATXN1[82Q] mice was used to evaluate expression of Ptch2 and Gli1 mRNA. Data is presented as mean ± SEM with average values for each mouse represented by a dot. N = 7, *p < 0.05 unpaired t test with Welch’s correction. (C) Ridgeplot showing distribution of Shh expression in hATXN1 + and hATXN1- PCs. P value adjusted using Benjamini-Hockberg method ≥0.05. [file Image_6.TIF]
